# Breast tumor cells promotes the horizontal propagation of EMT, stemness, and metastasis by transferring the MAP17 protein between subsets of neoplastic cells

**DOI:** 10.1038/s41389-020-00280-0

**Published:** 2020-10-26

**Authors:** José Manuel García-Heredia, Daniel Otero-Albiol, Marco Pérez, Elena Pérez-Castejón, Sandra Muñoz-Galván, Amancio Carnero

**Affiliations:** 1 Instituto de Biomedicina de Sevilla (IBIS), Hospital Universitario Virgen del Rocío, Universidad de Sevilla, Consejo Superior de Investigaciones Científicas, Seville, Spain; 2 Departamento de Bioquímica Vegetal y Biología Molecular, Universidad de Sevilla, Seville, Spain; 3 CIBER de Cancer, Seville, Spain

**Keywords:** Cancer stem cells, Breast cancer, Extracellular matrix

## Abstract

MAP17 (PDZK1IP1) is a small protein regulating inflammation and tumor progression, upregulated in a broad range of carcinomas. MAP17 levels increase during tumor progression in a large percentage of advanced tumors. In the present work, we explored the role of this protein shaping tumor evolution. Here we show that in breast cancer, cells increased MAP17 levels in tumors by demethylation induced multiple changes in gene expression through specific miRNAs downregulation. These miRNA changes are dependent on Notch pathway activation. As a consequence, epithelial mesenchymal transition (EMT) and stemness are induced promoting the metastatic potential of these cells both in vitro and in vivo. Furthermore, MAP17 increased the exosomes in tumor cells, where MAP17 was released as cargo, and this horizontal propagation also increased the EMT in the recipient cells. Importantly, an antibody against MAP17 in the media reduces the EMT and stemness alterations promoted by the conditioned media from MAP17-expressing cells. Therefore, MAP17 expression promotes the horizontal propagation of EMT and metastasis by transferring the MAP17 protein between subsets of neoplastic cells. Thus, MAP17 can be used to describe a new mechanism for cell malignity at distance, without the involvement of genetic or epigenetic modifications. MAP17 can also be taken in consideration as new target for metastatic high-grade breast tumors.

## Introduction

MAP17 (PDZK1IP1, DD96, SPAP)^1–3^ is a small (~17 kDa), non-glycosylated protein usually localized to the plasma membrane and associated with areas of cell–cell contact^4^. This protein has a C-terminal PDZ domain allowing the last four amino acids (STPM) to act as a carrier for transport from the Golgi to the cell membrane^5^. MAP17 interacts with PDZK1^6,7^, producing this relationship a variable complex that enables the transport of different molecules through the plasma membrane^8^.

Although its expression in non-tumor cells is restricted to specific epithelial cell populations in kidney^4^, >50% of advanced tumors exhibit significant expression of MAP17^1,3,8,9^. High MAP17 levels appear in most human carcinomas and in other non-epithelial neoplasias, such as glioblastomas or lymphomas^4,10^. Despite the relative scarcity of publications, MAP17 overexpression has been linked to a myriad of different effects. Thereby, its increased expression is associated with tumor progression, as it regulates both cellular transformation and malignancy^1,11,12^, being connected its expression with an increase in cell dedifferentiation^11–13^. In tumor cell lines with low MAP17 levels, its ectopic overexpression usually drives increased tumorigenic properties, such as stemness^13–15^. Further, MAP17 overexpression in tumor cells diminished apoptosis and induced increased growth of mouse tumors^10,13^. These data show that an increase in MAP17 correlates with an increase in the cancer stem cell (CSC)-like pool, regardless of the type of tumor cell^8,15^.

It has also been recently shown that the expression of MAP17 is not restricted to cancer, being also upregulated in chronic inflammatory diseases^16^. In fact, inflammation is a typical event in tumor progression^17^. MAP17 expression is correlated with filaggrin, a protein usually downregulated in inflammatory skin diseases, such as atopic dermatitis or psoriasis^18^. Moreover, MAP17 overexpression induces the transcription of genes related to inflammatory response, such as HLAs, IL-6, and NFAT2^8,16^. These data suggest that the upregulation of MAP17 is an important event both in cancer and inflammatory diseases.

Although MAP17 has no enzymatic or transcriptional activity, it exerts its described roles through the regulation of several cell signaling pathways. MAP17 overexpression activates Notch pathway by sequestrating NUMB, a known inhibitor of NOTCH, allowing a higher activation of the pathway^15,19^. Also, MAP17 overexpression reduces NFκB activation and cell autophagy by increasing reactive oxygen species (ROS)^20^. Thus, MAP17 has been connected with a 30–40% increment in ROS^11,13,14^. It has been previously described that altered ROS levels promote various pathological conditions, including cancer, through changes in gene expression regulation, increased mutagenic rates and genomic instability^9,11^.

Exosomes, and other extracellular vesicles (ExVs), have attracted the attention of researchers, owing, on the one hand, to their potential as biomarkers and, on the other, to their ability to modify the behavior of the cells that receive them^21–24^. Exosomes can provide a variety of extracellular environments for cellular communication, and modify the conditions to adapt to different physiological settings^24^. The role of exosomes in EMT modification and metastasis growth during tumorigenesis has been largely proven^25–27^. These structures are small ExVs that can be secreted by a variety of normal, immune, or tumor cells^27^. By packaging different compounds such as RNAs, proteins, lipids, or signaling molecules, they can participate in cell to cell communication^22,24^. Meanwhile, MAP17 has been found to increase with tumor stage^1,11,12^ and increased EMT properties^4,9–11^, being also related to immune attraction to tumor site^8,16^. These properties of MAP17 seem to fit well with the reported role for the exosome-dependent adaptations. Therefore, in the present work we explored whether MAP17 provides any exosome-dependent microenvironment adaptation, through exosome release, with relevance in tumor metastasis. To this end, we measured whether MAP17-induced EMT and metastatic properties are dependent on exosomes release and which is the mechanism for such behavior.

One of the elements usually included inside exosomes and other ExVs are microRNAs (miRNAs)^28,29^. These short non-coding RNAs regulate the post-transcription expression of multiple target genes being, as consequence, known as broad modulators of gene expression^30^. Among the known functions of miRNAs are both modulations of EMT and metastasis^31,32^. As MAP17 produce broad transcriptional effects^11,13–15,20^, compatible with changes in miRNAs expression, we decided to look for coordinated changes in miRNAs expression owing to MAP17 expression.

Here, we found that MAP17 overexpression could exert part of its broad effects as a physiological miRNA modulator, turning MAP17, a commonly overexpressed gene in carcinomas, into a master regulator of cell dedifferentiation. MAP17 protein increases EMT, stemness and metastasis in vivo. In addition, part of the alterations found on miRNA expression was dependent on the activation of the Notch pathway. This increase in MAP17 expression induced also an increment in ExVs release, which propagates MAP17 between subsets of neoplastic cells. Thus, the horizontal transference of MAP17 protein promotes EMT and stemness in target cells not overexpressing MAP17. Finally, the elimination of MAP17 by antibodies from the media reduces this increase in EMT and stemness, suggesting that MAP17 could be a new target for metastasis in advanced tumors.

## Materials and methods

### Bioinformatics analysis

To establish a possible connection between *MAP17* levels and tumor progression, we used Finak data set (GSE9014) from Oncomine (https://www.oncomine.org). In addition, we used R2 webpage resource to compare MAP17 expression across data sets, using 219630_at as probe for MAP17 and the algorithm MAS5.0 for data normalization (see Supplementary Table 1). Kaplan–Meier method was used for survival analysis, according to R2 webpage adjustments. TCGA Wanderer resource (data sets for Breast Invasive Carcinoma, Colon Adenocarcinoma and Lung Adenocarcinoma) was used to analyze the methylation state of *MAP17* in human samples^33^, considering CG probes cg15187606 and cg26523175, both upstream of MAP17 gene.

To find genes correlated with *MAP17* expression, we selected 31 breast cancer databases (see Supplementary Table 1), all freely accessible through R2 webpage (http://r2.amc.nl). We used two different gene filters: Oncogenesis (GeneCategory) and Pathways in Cancer (KEGG Pathway); both options included in R2. We searched for correlations using the *MAP17* probes listed in Supplementary Table 1, establishing a *p* value < 0.05 to identify significant differences. From the list of correlated genes, we separated genes positively from genes negatively correlated with *MAP17* expression, generating two gene lists for each database.

To look for altered biological processes connected to changes in *MAP17* expression, we used enrichment analysis from Gene Ontology consortium webpage (http://geneontology.org/page/go-enrichment-analysis). The obtained GO terms, from genes that were either positively or negatively correlated with MAP17 expression, were compared using Venny tool^34^. In addition, we used Panther (http://www.pantherdb.org/) to group the list of genes according to protein class.

TransmiR v2.0 software (http://www.cuilab.cn/transmir) was used to find miRNAs regulated by NOTCH1, HES1, or HES5.

Data sets GSE20685 and GSE7390 were used to separate patients according to tumor type (primary vs metastasis) and *MAP17* levels (low vs high), using GEO2R (https://www.ncbi.nlm.nih.gov/geo/geo2r/) to obtain the expression values of each individual gene.

### Cell lines and cellular assays

T-47D, MDA-MB-231, MDA-MB-468, and MCF10A cells were obtained from the European Collection of Authenticated Cell Cultures (ECACC) commercial repository at the beginning of this study. No further authentication was performed in these cell lines. AA, AW, AX, BC, and CE cell lines, derived from sarcoma patients, were described previously^35^. T-47D, MDA-MB-231, and MDA-MB468 cells were maintained in DMEM (Gibco), whereas sarcoma cells were maintained in F10 (Gibco), all supplemented with 10% fetal bovine serum (FBS; Life Technologies), penicillin, streptomycin, and fungizone. All cell lines were regularly tested for mycoplasma. MAP17 expression was induced through transfection with plasmid pBabe-MAP17, previously described^12,15^. All transfected cells were selected with 1 μg mL^−1^ of puromycin. Clonogenicity assays, holo- and paraclone analysis and tumorspheres analysis were performed as previously described^36^.

### miRNAs analysis

We extracted total RNA from T-47D cells, overexpressing MAP17 or control, using Qiazol and miRNAeasy kit (Qiagen, USA). To find miRNAs with significant differences between both conditions, we used the Cancer Pathway Finder miScript miRNA PCR Array (Qiagen, USA), following manufacturer’s instructions. All miRNAs detected with significant differences were analyzed using miRTarBase resource (miRTarBase.mbc.nctu.edu.tw/), focusing only in changes in gene expression detected by direct Reporter Assay or Western Blot.

### Analysis of gene transcription

Total RNA was purified as described previously^15,36^. To detect changes in gene expression, we used the probes listed in Supplementary Table 2. From the list of miRNAs with significant changes, we selected five of them, also listed in Supplementary Table 2. All probes were purchased from Life Technologies and retro-transcribed following manufacturer’s instructions.

Quantitative PCR was performed as described previously^15,36^. At least three independent experiments in triplicate samples were performed for each analyzed gene. Student’s *t* test was applied for each pair of samples, with a significance threshold of *p* < 0.05.

### Protein extraction and WB analysis

Protein extracts for western blot (WB) analysis were obtained as described previously^36^, with the exception of cell extracts used for MAP17 detection, where adioimmunoprecipitation assay (RIPA) buffer included 6 M urea. For WB detection, we used anti-MAP17 monoclonal antibodies, anti-SNAI1 (Cell Signaling, C15D3), anti-CDH1 (Santa Cruz Biotechnology, sc-8426), anti-CDH2 (Santa Cruz Biotechnology, sc271386), anti-CD63 (Thermofisher #10628D), and anti-calnexin (Santa Cruz, sc-23954). α-Tubulin (T9026, Sigma) was used as a control. Horseradish peroxidase-labeled rabbit anti-mouse (ab97046, Abcam) and goat anti-rabbit (ab97051, Abcam) secondary antibodies were used.

### Fluorescence-activated cell sorting (FACS) analysis

MDA-MB-231, MDA-MB-468, T-47D, AA, AW, AX, BC, and CE cells were washed once with PBS and harvested with 0.05% trypsin/0.025% EDTA. Detached cells were centrifuged and resuspended in wash buffer (PBS, 2% FBS, 5 mM EDTA). One million cells was resuspended in 125 μL of this buffer and blocked for 10 minutes with 12.5 μL of FcR Blocking Reagent (MACS MiltenyiBiotec, 130–059–901). Combinations of fluorochrome-conjugated monoclonal antibodies from MACS MiltenyiBiotec against human CD44 (APC; 130–095–177), CD24 (PE; 130–095–953); CD63 (APC; 130–100–182), CD105 (APC; 130–099–125) or CD133 (PE; 130–098–826) were added to the cell suspension following manufacturer’s instructions and incubated at 4 °C in the dark for 30 minutes. Labeled cells were washed twice with wash buffer, resuspended in 300 μL of wash buffer and analyzed on a FACS Canto II Analyzer cytometer.

### Cell migration and invasion assay

Cells (2.5 × 10^5^) were seeded in Boyden chambers with 8.0 µm pore size (Nunc, Thermo Fisher) in serum-free medium. Medium containing 10% FBS served as a chemoattractant in the lower chamber. After 8 h, cells were fixed with glutaraldehyde 0.5% and stained with crystal violet 1%. Next, non-invading cells were removed with cotton swabs. Ten microscopic fields of invading cells were counted for each well. Data are represented as the mean ± SEM from three individual experiments.

### Mouse luciferase assay

To determine whether MAP17 increases metastatic potential, MDA-MB-231 cells, previously transfected with pBabe-EV or pBabe-M17, were infected with plasmid pLenti-II-CMV-Luc2-IRES-GFP, which allow expression of both luciferase and GFP, and GFP^+^ cells were selected by flow cytometry. Three million cells from each cell line were injected into the mammary fat pad of three six-week-old Foxn1^nu^ athymic nude female mice (Harlan Laboratories, Netherlands), with no randomization nor researcher blinding. When primary tumors reached a size of 10 mm^2^, tumors were surgically removed and mice continually assessed for tumor recurrence. To analyze the appearance of metastatic tumors, a luciferin solution (XenoLight D-luciferin-K^+^ salt bioluminescent substrate, PerkinElmer, 122799) was injected at a final concentration of 150 mg luciferin/kg of mouse body weight. Mice were anesthetized with inhaled isoflurane 10 minutes after luciferin injection. To detect GFP fluorescence, mice were analyzed 15 minutes after luciferin injection in an IVIS Lumina Series III (Perkin Elmer). Then, mice were killed with CO_2_, and their organs were also visualized. In all cases, the optimal exposure time was determined by the software (Living Image 4.5.4).

### Treatment with conditioned media

Conditioned media from transfected MDA-MB-231 and MDA-MB-468 cells were obtained and used as previously described^16^. Non-transfected MDA-MB-231, MDA-MB-468, T-47D, AA, AW, AX, BC, and CE cells were seeded in six-well plates and allowed to grow for 24 hours. After that, media was substituted by a 1:1 conditioned media:fresh medium, and cells grow for other 48 hours before total RNA was extracted to evaluate changes in miRNAs, gene expression and surface cell markers, as described above. In addition, in order to evaluate if vesicles derived from conditioned media were responsible for cell dedifferentiation, 4 mL of conditioned media were incubated with 5 μL of aldehyde/sulfate latex beads Beads 4% w/v (Thermo Fisher, A37304) under stirring for 2 hours. After that, media was centrifuged at 4000 rpm for 5 minutes, using supernatant to treat MDA-MB-231 and MDA-MB-468 cells for 48 hours, in a proportion 1:1 with fresh medium. For tumorsphere assay, a total of 10^5^ cells were cultured in Ultra-Low Attachment Multiwell Plates (Corning) in triplicate, and tumorspheres were counted 5 days after seeding.

In addition, 4 mL of conditioned media were incubated with 100 μL of polyclonal antibodies against MAP17 and 10 μL of protein A-sepharose or only with 10 μL of protein A-sepharose under stirring for 2 hours at 4 °C and centrifuged as above. As a control, 4 mL of conditioned media, previously treated with 5 μL of aldehyde/sulfate latex beads 4% w/v for 2 hours and centrifuged as above, were incubated also with 100 μL of polyclonal antibodies against MAP17 and 10 μL of protein A-sepharose. All conditioned media were used for treating MDA-MB-231 cells for 48 hours and total RNA was extracted as described above.

### ExVs isolation and detection

Conditioned media from MDA-MB-231 or MDA-MB-468 cells were centrifuged for 30 min at 10,000 × g at 4 °C, transferred to a new tube, equilibrated with TBS-Ca^2+^ and centrifuged 2 hours at 100,000 × *g* at 4 °C. Then, the supernatant (SN) was discarded, TBS-Ca^2+^ was added again, and the sample was centrifuged for 1 hour at 100,000 × *g* and 4 °C. Finally, the SN was discarded by decantation, being the pellet (considered to be an ExVs-enriched fraction) resuspended in RIPA buffer for detection by WB.

To detect ExVs fusion in cells, MDA-MB-231 or MDA-MB-468 cells (EV or MAP17) overexpressing cytoplasmic GFP were cultured for 72 hours, and ExVs were purified as indicated above. Non-transfected MDA-MB-231 cells, seeded in six-well plates, were used to detect possible ExVs in cells. For that purpose, an ExVs-enriched fraction, resuspended in TBS-Ca^2+^ buffer, was added to the cells and incubated at 37 °C, 5% CO_2_ for 90 minutes. After that, cells were visualized using an Olympus BX61 fluorescence microscope.

For FACS analysis of ExVs, 3.5 × 10^5^ MDA-MB-231 or MDA-MB-468 cells were seeded at 6 cm^2^ plates with 3 mL of DMEM and cultured for 48 hours. Then, cell media was removed and centrifuged for 30 min at 10,000 × *g* at room temperature, being the supernatant incubated with 2 µL of aldehyde/sulfate latex beads 4% w/v (Thermo Fisher, A37304) for 30 min at room temperature in a rotator mixer. Finally, the beads were recovered by centrifugation at 4000 × *g* for 5 minutes, washed twice with PBS, and labeled with CD63 antibody using the conditions described above.

### Ethics approval

All methods were performed in accordance with the relevant guidelines and regulations of the Institute for Biomedical Research of Seville (IBIS) and University Hospital Virgen del Rocio (HUVR). Animal experiments were performed according to the experimental protocol approved by HUVR Animals Ethics (CEI 0309-N-15).

### Availability of supporting data

No data sets were generated during the current study. The data sets analyzed during the current study are available in the different repositories already mentioned in the Methods section.

## Results

### MAP17 is a common feature of human metastatic tumors

**Fig. 1:**
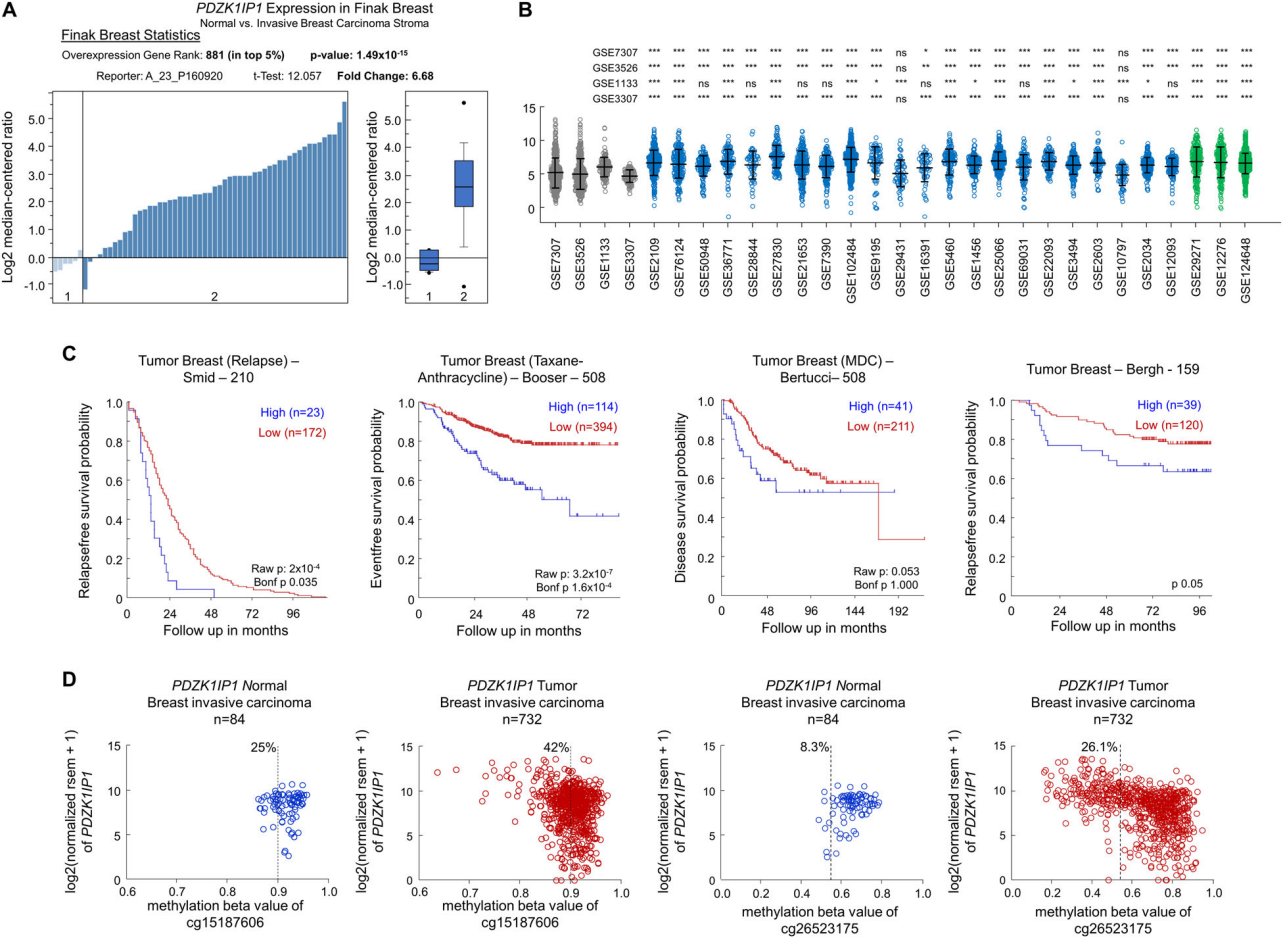
MAP17 upregulation is a common event in breast tumor progression. **a** *MAP17* (*PDZK1IP1*) expression levels are higher in tumor samples than normal samples in the Finak Breast data set (1. normal tissue (*n* = 6); 2. invasive breast carcinoma (*n* = 53)). **b** MAP17 expression across data sets, showing increased expression in breast tumors regarding normal tissues (gray: normal data sets; blue: primary breast cancer data sets; green: metastatic breast data sets). Student’s *t* test statistical analysis of the data was performed to find significant differences (*p < 0.05; **p < 0.01; ***p < 0.001). **c** MAP17 upregulation is associated with worse survival in breast tumor patients. **d** Tumor breast samples exhibit a lower methylation state of the *PDZK1IP1* gene, which is most likely correlated with its deregulation.

To determine whether MAP17 can increase the metastatic potential of human tumors, we looked for changes in MAP17 expression in breast data sets. We observed that *MAP17* levels were significantly higher in tumor samples than in normal samples (Fig. 1a, b), being also significantly increased in metastatic samples (Fig. 1b). In addition, patients with high levels of *MAP17* had worse prognosis and lower survival rates than patients with lower *MAP17* levels (Fig. 1c). Both results suggest that *MAP17* expression could be deregulated in breast tumors, so we analyzed possible changes in *MAP17* methylation state. Using two CpG probes upstream of the coding sequence, a significant number of tumor samples showed lower methylation beta values than normal samples (Fig. 1d). In tumor breast samples, demethylation of *MAP17* gene can increase its transcription in a subset of the tumor cell population, increasing its tumorigenic properties^1,4,15^. As a consequence of this possible deregulated expression state, we decided to investigate the role of *MAP17* increase in breast tumor progression.

### MAP17 expression correlates with changes in gene expression and modulates miRNA expression

**Fig. 2:**
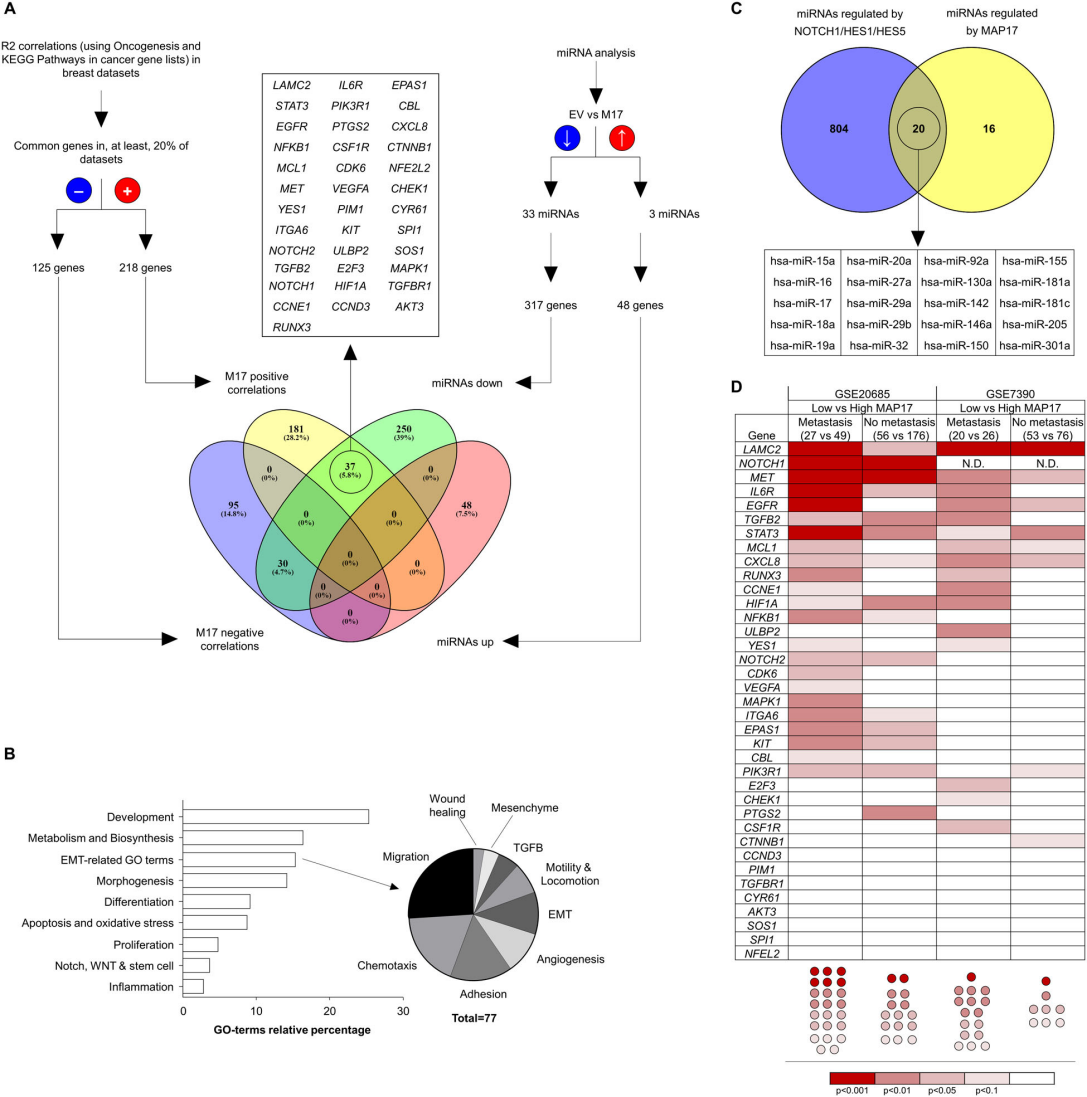
MAP17 modulates changes in gene expression in breast tumors. **a** Flow chart of the strategy used to identify genes modulated by MAP17 in breast tumor data sets or T-47D tumor cells due to changes in the miRNA population; a list of 37 genes connected both to miRNA downregulation and MAP17 positive correlation is shown. **b** A diagram is shown of the most common GO terms obtained from the 37 gene list, showing processes previously associated with MAP17 overexpression, such as oxidative stress and the Notch pathway. EMT-related GO terms appear to be a prominent group, composed of a heterogeneous group of terms, including migration, EMT, adhesion and wound healing. **c** Twenty of the identified miRNAs are regulated by NOTCH1, HES1, or HES5. **d** The identified genes from our screen are mainly significantly different in metastatic samples from breast human tumors.

*MAP17* overexpression has been previously connected with cell dedifferentiation^1,14,15^, a critical step in acquiring CSC properties. Therefore, we decided to assess the correlation between *MAP17* levels and specific genes connected to tumor progression in breast cancer. Using R2 software, we looked for changes in the expression of genes connected to tumorigenesis and altered pathways in cancer. We found a total of 218 and 125 genes whose expression was positively or negatively correlated with *MAP17*, respectively (Supplementary Table 3). A significant number of these genes, both those that were positively and negatively correlated with MAP17, were transcription factors, according Panther analysis (Supplementary Fig. 1). This result suggests that MAP17 could modify the expression of a high number of genes, causing pleiotropic effects and modifying different pathways in cancer. In fact, MAP17 has been found to interact with NUMB, allowing activation of Notch pathway^15^, which exhibits crosstalk with miRNAs^30,37^, appearing the last commonly related to changes in gene translation^30,38^. Owing to the previously described role of MAP17 in tumors^1,8,9,15^ and the high prevalence of MAP17 in breast cancer, we analyzed changes in miRNA population, finding a total of 36 miRNAs with significant differences between parental and MAP17-overexpressing cells (Supplementary Table 4). Most of these miRNAs (33 of 36) were downregulated, allowing an increase in the levels of their targets. Each individual miRNA was analyzed using miRTarBase to identify only validated miRNA targets, finding 48 and 1117 genes modulated by the upregulated and downregulated miRNAs, respectively (Supplementary Table 4). For downregulated miRNAs, owing to the higher number of target genes, we selected those genes modulated by at least two different miRNAs, obtaining a list of 317 genes. To gain insight into which genes were altered by MAP17 through miRNAs, we established connections between genes correlated with *MAP17* expression and genes modulated by the downregulated miRNAs in cells overexpressing MAP17; we found 37 genes corresponding to downregulated miRNAs and MAP17 positive correlations (Fig. 2a). By using enrichment analysis from Gene Ontology, we identified a total of 918 GO terms of biological process (Supplementary Table 4), being a significant number of them directly related to stem cells, Notch pathway and EMT-related genes (Fig. 2b). In addition, we found that up to 20 of the found miRNAs were regulated by the transcription factors NOTCH1, HES1, or HES5, suggesting a clear connection between MAP17 overexpression and miRNAs regulation through Notch pathway activation (Fig. 2c).

To analyze the genes modulated both by miRNA downregulation and MAP17 overexpression, we examined the expression pattern of the 37 identified genes in human tumor breast data sets. By doing this, and by separating tumor samples by their origin (primary vs metastatic tumor) and *MAP17* expression (low vs high), we found a clear trend in this list; appearing small differences according the data set considered. We found that, although some genes appeared to be significantly different in both primary and metastatic tumors (*LAMC2*, *NOTCH1*, *MET*, *IL6R, or STAT3*), a significant percentage of the genes only had significant differences in metastatic tumors (Fig. 2d), suggesting that high MAP17 expression induces changes in cell expression that potentiates differentiation toward EMT and/or CSC phenotypes.

### MAP17 causes miRNA downregulation and EMT in breast cancer cells

**Fig. 3:**
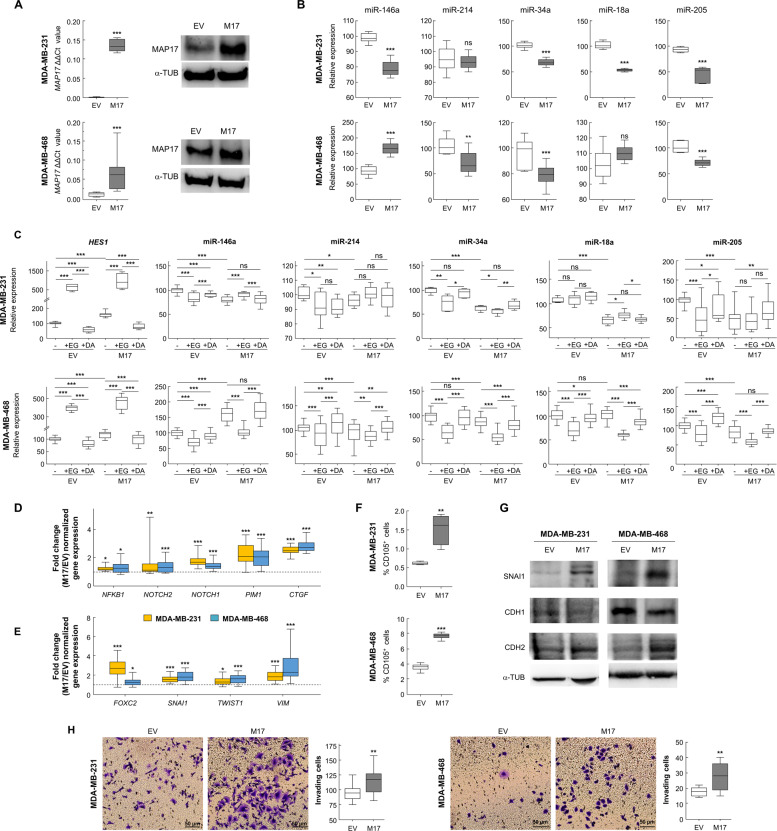
MAP17 overexpression induces miRNA expression that drives EMT. **a** MAP17 expression levels, measured both by RT-qPCR and WB, are shown in transfected breast cancer cells. **b** MAP17 overexpression reduces the levels of five of the miRNAs identified in our screening. **c** Changes in transcriptional levels are shown for *HES1* and miRNAs owing to Notch pathway activation by EGTA (EG) or inhibition by DAPT (DA) treatments. **d** Fold change are shown for five genes targeted by some of the identified miRNAs, upregulated in cells overexpressing MAP17. **e** Fold change of EMT-related genes in breast cells overexpressing MAP17 is shown. **f** Analytic FACS was used to identify the CD105^+^ subpopulation in MDA-MB-231 or MDA-MB-468 cells. **g** WB of SNAI1, CDH1, CDH2, and α-tubulin in MDA-MB-231 and MDA-MB-468 is shown, where MAP17 overexpression modifies the expression of proteins involved in EMT. **h** Boyden chamber assay results are shown, observing that MAP17 overexpression causes an increase in invasive ability. Student’s *t* test statistical analysis of the data was performed to find significant differences (*p < 0.05; **p < 0.01; ***p < 0.001). *EV* empty vector; M17, MAP17.

To verify both bioinformatics and miRNA results, we used two other breast tumor cell lines, MDA-MB-231 and MDA-MB-468, transfected to overexpress MAP17, as assessed by both mRNA and protein levels (Fig. 3a).

Then, we focused on the previously identified GO-term connection between MAP17 and EMT (Fig. 2). We determined that MAP17 overexpression caused a significant downregulation of the five miRNAs tested in MDA-MB-231, three in the case of MDA-MB-468 cells, appearing only miR-146a in MDA-MB-468 cells upregulated in cells overexpressing MAP17, suggesting in this case a probably cell-specific regulation (Fig. 3b).

Owing to the previously described interaction of MAP17 with NUMB^15^, an inhibitor of Notch pathway, we analyzed both the activation and inhibition of this pathway in both breast cancer cell lines. As a control for this experiment, we used a known target of Notch pathway, *HES1*. We detected higher basal activation of *HES1* transcription in cells overexpressing MAP17, as we previously described^15^, showing that MAP17 activates Notch pathway (Fig. 3c). In addition, EGTA treatment induced *HES1* transcription. On the other hand, inhibiting Notch pathway by DAPT, a known inhibitor of Notch pathway, reduced *HES1* transcription, indicating its dependence of Notch pathway activation. Regarding the miRNAs, we found a general decrease in their expression when Notch pathway was activated with EGTA. In addition, when treating the cells with DAPT, the levels of miRNAs expression were recovered to the parental cell line (EV or MAP17) levels. However, differences in expression between EV and MAP17 remain, besides EGTA or DAPT treatments. (Fig. 3c). These results suggest a regulation of these MAP17-regulated miRNAs through the Notch pathway. In addition, we measured the expression of some of the genes found when comparing miRNA levels and MAP17 expression. In all cases, we found a significant increase in gene transcription (Fig. 3d, Supplementary Fig. 2A).

To test the possible role of MAP17 in EMT, we analyzed the expression of genes that our bioinformatics analysis found to be connected to EMT. MAP17 overexpression upregulated the transcription of *FOXC2*, *SNAI1*, *TWIST1,* and *VIM*, genes previously found to be related to EMT (Fig. 3e, Supplementary Fig. 2B). To look for other typical EMT features, we analyzed CD105 expression, described as a specific mesenchymal marker^39^, finding a significant increase in the CD105^+^ population owing to MAP17 overexpression (Fig. 3f). We also found that cells overexpressing MAP17 showed reduced expression of CDH1 and higher CDH2 and SNAI1 levels, as determined by WB (Fig. 3g).

Finally, to analyze changes in the invasive properties of cells overexpressing MAP17, we performed a transwell invasion assay, finding an increased percentage of invasive cells when MAP17 was overexpressed (Fig. 3h).

Therefore, our data show an increase in EMT properties due to MAP17 activities in breast tumor cells.

### MAP17 overexpression increases tumor-initiating potential in breast cancer cells

**Fig. 4:**
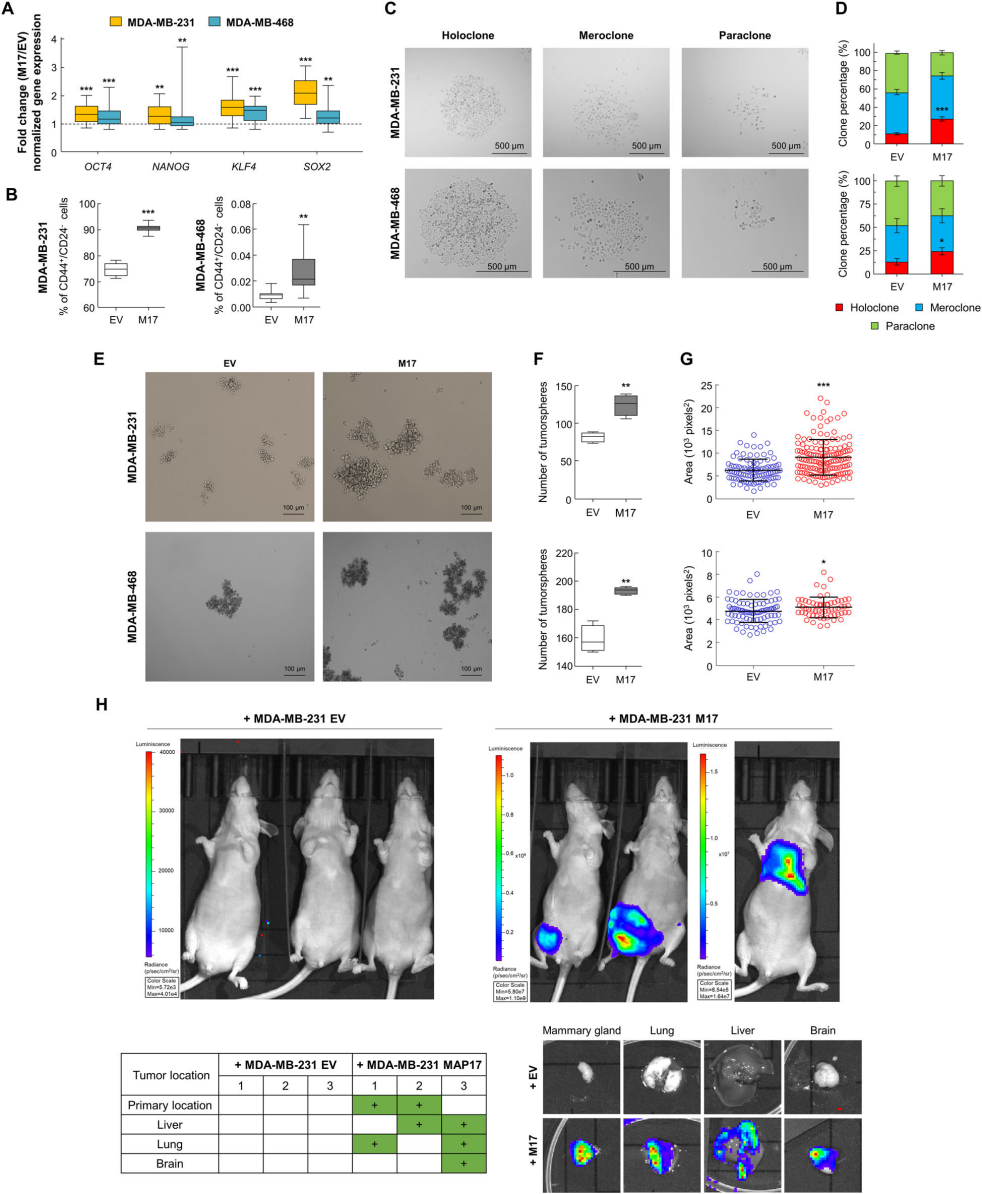
MAP17 overexpression increases CSC properties and metastatic potential in breast cancer cells. **a** Fold change of CSC markers (*KLF4*, *NANOG*, *OCT4,* and *SOX2*) in MDA-MB-231 and MDA-MB-468 cells, showing an increase due to MAP17 overexpression. **b** Analytic FACS was used to identify the CD44^+^/CD24^−^ subpopulation in MDA-MB-231 or MDA-MB-468 cells. **c** Bright-field microscopy images are shown of a typical holoclone, meroclone, or paraclone from MDA-MB-231 or MDA-MB-468 cells. **d** MAP17 overexpression causes an increase in holoclones percentage. The percentages of each type of clone are shown. At least 150 individual clones were analyzed in triplicate. **e** The morphology of tumorspheres derived from MDA-MB-231 and MDA-MB-468 cells is shown. **f** Number of tumorspheres obtained from MDA-MB-231 or MDA-MB-468 cells is shown. **g** The area of the tumorspheres generated from MDA-MB-231 or MDA-MB-468 cells is shown. MAP17 overexpression induces a higher number of tumorspheres and with a larger size, in both cell lines. **h** Cells overexpressing MAP17 induce metastases in nude mice. Colored regions in the mice or their organs correspond to transfected cells expressing luciferase. Student’s *t* test statistical analysis of the data was performed to find significant differences (*p < 0.05; **p < 0.01; ***p < 0.001). *EV* empty vector; M17, MAP17.

CSCs, proposed to be the true metastatic initiating cells, are characterized by increased expression of stem cell factor genes, such as *OCT4*, *NANOG*, *SOX2* and *KLF4*^40,41^. Importantly, *NANOG*, *SOX2,* and *KLF4* are described as downregulated by at least three of the identified miRNAs, being *OCT4* described as downregulated by only one miRNA (Supplementary Table 5). Therefore, we looked for changes in the expression of these CSC markers in both breast cancer cell lines overexpressing MAP17, observing significantly increased mRNA levels of all these stem cell transcripts (Fig. 4a, Supplementary Fig. 2C). To confirm the cancer stem-like phenotype of MAP17-expressing cells, we analyzed the cellular subpopulations expressing CSC surface markers. Both MDA-MB-231 and MDA-MB-468 are mammary tumor cells, and the CSC subpopulation of each is described as CD44^+^/CD24^−^^42^. Therefore, we measured these subpopulations, finding that cells overexpressing MAP17 contained a significantly larger CD44^+^/CD24^−^ population than what was observed in the EV cells (Fig. 4b).

To analyze the previously described positive impact of increased MAP17 levels on the CSC phenotype^15^, we measured both clonal growth and the formation of tumorspheres, commonly associated with the cancer-initiating cell phenotype and the ability to generate new colonies. The first experiment allowed us to distinguish between holoclones (colonies derived from CSCs) from paraclones (colonies derived from differentiated cells incapable of reconstituting a culture) and meroclones (those with intermediate properties)^43,44^. The different types of colony morphologies allow us to predict some stem cell characteristics^43^. Therefore, we measured whether MAP17 overexpression increases the percentage of holoclones in both breast cancer cell lines, observing a significant increase in the percentage of holoclones owing to MAP17 overexpression (Fig. 4c, d).

Human tumor cell populations can be maintained in serum-free suspension cultures allowing their growth as clusters of cells called “tumorspheres”^45^. These tumorspheres are enriched with multipotent epithelial progenitors^40^ with higher expression of CSC markers^41^. Therefore, we measured the ability of tumor cancer cell lines with modified MAP17 expression to form tumorspheres. EV or MAP17-overexpressing cells were subjected to disaggregation and seeded in tumorsphere media, allowing forming tumorspheres for 5–7 days (Fig. 4e). For both breast cancer cell lines, both the number and size of tumorspheres were significantly increased in MAP17-overexpressing cells (Fig. 4f, g).

Finally, to verify the whole metastatic phenotype was induced by MAP17, we analyzed whether this increase in EMT and stemness properties was also connected with an increased metastatic potential in vivo. In this way, mice transfected with breast cells overexpressing MAP17 were injected into the mammary fat pad and extracted when the tumors reached 1 cm^3^. After 2 months, only cells with increased MAP17 levels showed both tumor recurrence and metastasis in, at least, liver, lung, and brain (Fig. 4h).

### MAP17 induces increased ExVs secretion

**Fig. 5:**
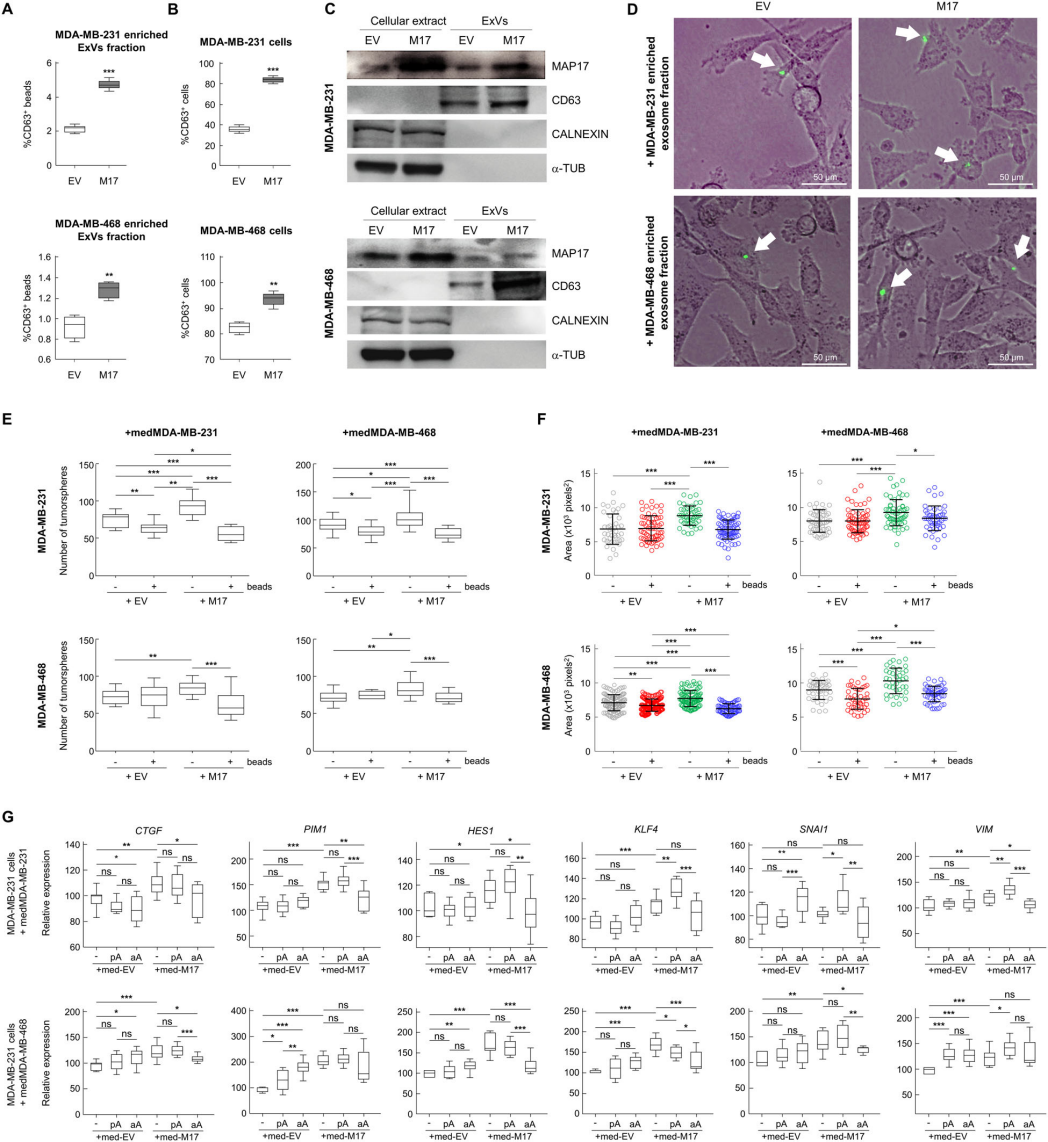
MAP17 overexpression induces increased secretion of ExVs. **a** Analytic FACS was used to identify the CD63^+^ subpopulation in the ExVs-enriched fraction purified from the conditioned media of MDA-MB-231 or MDA-MB-468 cells; ExVs were detected by binding to aldehyde/sulfate latex beads. **b** Analytic FACS was used to identify the CD63^+^ subpopulation in MDA-MB-231 or MDA-MB-468 cells. **c** Detection of ExVs in the supernatants from MDA-MB-231 and MDA-MB-468 cells with or without MAP17 overexpression was performed by WB. CD63 was used as an ExVs marker, and α-tubulin and calnexin were used as negative controls (nuclear and RE markers, respectively). Anti-MAP17 antibody was also used to detect this protein in ExVs. Total cellular extracts of both breast cell lines overexpressing MAP17 or carrying EV were used as controls. **d** Detection was performed for the detection of possible ExVs derived from MDA-MB-231 cells grown in MDA-MB-231 or MDA-MB-468-conditioned media. White arrows mark possible ExVs labeled with GFP. **e** Number of tumorspheres from MDA-MB-231 or MDA-MB-468 incubated with conditioned or deconditioned media derived from EV or MAP17-overexpressing cells. **f** Size of tumorspheres from MDA-MB-231 or MDA-MB-468 incubated with conditioned or deconditioned media derived from EV or MAP17-overexpressing cells. **g** Treatment of MDA-MB-231 cells with conditioned media derived from MDA-MB-231 and MDA-MB-468 cells, treated with antibodies against MAP17 (− conditioned media, *pA* conditioned media plus protein A-sepharose, *aA* conditioned media plus antibodies against MAP17 and protein A-sepharose). Student’s *t* test statistical analysis of the data was performed to find significant differences (*p < 0.05; **p < 0.01; ***p < 0.001). *EV* empty vector; M17, MAP17.

Our previous results suggest that MAP17 activates a secretory phenotype inducing cell dedifferentiation. Therefore, we analyzed the presence of ExVs from both MDA-MB-231 and MDA-MB-468 cells purifying them by coupling to aldehyde/sulfate latex beads and labeling them with a CD63^+^ antibody. This allowed us to detect an increase in the ExVs population in MAP17-overexpressing cells (Fig. 5a). In addition, breast cells overexpressing MAP17 also showed an increase in CD63^+^ population (Fig. 5b). This result indicates an increase in ExVs secretion from MAP17-expressing cells. We also purified ExVs, finding that not only had higher CD63^+^ protein levels but were also loaded with higher amounts of MAP17 protein (Fig. 5c). The absence of calnexin and tubulin suggests that purified ExVs are absent from endoplasmic reticulum or cytoskeleton. These purified ExVs, which were labeled with GFP, were observed inside non-transfected cells, showing the possibility that these ExVs entered in target cells, inducing dedifferentiation through MAP17 protein-mediated pathways (Fig. 5d). In addition, we co-cultured non-cancerous breast cell line MCF10A with MDA-MB-231 labeled with GFP, observing both an increment of fluorescent structures, compatibles with ExVs, in MAP17-overexpressing cells (Supplementary Fig. 3A). Some of these structures appear over MCF10A, suggesting a possible fusion of them with non-transfected cells (Supplementary Fig. 3B). In order to test if cell dedifferentiation was due to ExVs or other soluble factors from conditioned media, we eliminated ExVs from these media with aldehyde/sulfate latex beads, obtaining the so-called “deconditioned media”. We treated both MDA-MB-231 and MDA-MB-468 cells with conditioned or deconditioned media for 48 hours and, after that, we seeded in low attachment plates to form tumorspheres. After 5 days, we observed both smaller size (Fig. 5e) and lower number (Fig. 5f) of tumorspheres derived from deconditioned media, suggesting that factors contained in ExVs, like MAP17, were the responsible of cell dedifferentiation.

In order to evaluate if MAP17 has a direct role in this transforming process, we added antibodies against MAP17 to conditioned media. In these conditions, we observed that, for conditioned media derived from both MAP17-overexpressing cell lines, the treatment with anti-MAP17 antibodies abrogated gene upregulation caused in target cells by cell exposition to MAP17-derived conditioned media (Fig. 5g, Supplementary Fig. 4). These data suggest that MAP17, by itself, is capable to induce cell dedifferentiation epigenetically, by horizontal transference.

### Conditioned media derived from MAP17-overexpressing cells induces cell dedifferentiation

Although not fully understood, the roles of intercellular communication between cells are important^46^, especially in the establishment of the premetastatic niche, the promotion and growth of distant tumor cells and the changing of the immune system^47–49^. Therefore, ExVs can act as important regulators in metastatic cascades by delivering functional molecules and affecting target cells.

**Fig. 6:**
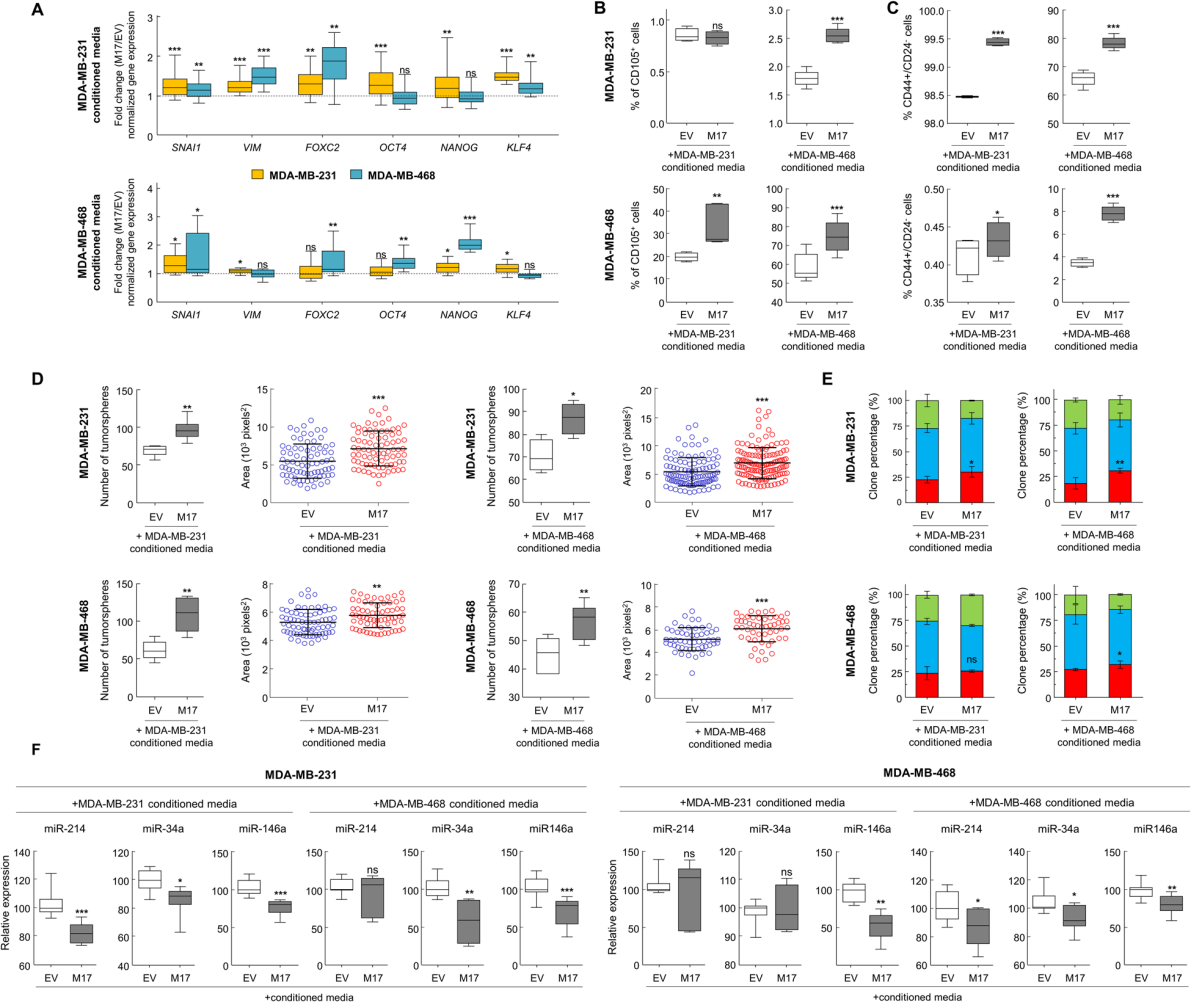
Conditioned media from MAP17-overexpressing cells induce cell dedifferentiation. **a** Media from MAP17-overexpressing cells induce changes in the transcriptional levels of genes connected both to EMT and CSC in MDA-MB-231 and MDA-MB-468 non-transfected cells. **b** Analytic FACS was used to identify the CD105^+^ subpopulation of MDA-MB-231 or MDA-MB-468 cells incubated with conditioned media. **c** Analytic FACS was used to identify the CD44^+^/CD24^−^ subpopulation of MDA-MB-231 or MDA-MB-468 cells incubated with conditioned media. **d** Number and area of tumorspheres in MDA-MB-231 or MDA-MB-468 cells treated with conditioned media derived from MDA-MB-231 or MDA-MB-468 cells is shown. **e** The percentages are shown for holoclones, meroclones, and paraclones in MDA-MB-231 or MDA-MB-468 cells treated with conditioned media derived from MDA-MB-231 or MDA-MB-468 cells. **f** Breast tumor cells incubated with MAP17-conditioned media exhibited lower levels of the three selected miRNAs identified in our screening. Student’s *t* test statistical analysis of the data was performed to find significant differences (*p < 0.05; **p < 0.01; ***p < 0.001). *EV* empty vector; M17, MAP17.

To analyze the possible mechanism by which MAP17 induces changes and EMT in non-transfected cells, especially in light of its previously described role in inflammation^16^, we tested whether conditioned media derived from MAP17-expressing MDA-MB-231 or MDA-MB-468 cells could affect the properties of either parental breast cell line. We found that cells incubated with MAP17-conditioned media exhibited changes in genes connected both to EMT and the acquisition of CSC properties (Fig. 6a, Supplementary Fig. 5A, B). Furthermore, we observed an increase in the number of both CD105^+^ cells (Fig. 6b) and CD44^+^/CD24^−^ cells (Fig. 6c), confirming that treatment with conditioned media alone induces dedifferentiation of breast tumor cells. To test whether this effect was general, we confirmed the results in T-47D cells and in several sarcoma primary cell lines, where we observed a general increase in the CD105^+^ population, showing activation of EMT in a significant percentage of the cells (Supplementary Fig. 6A, B). In addition, we found a significant increase in the population of cells with CSC-like properties owing to the increment in CD44^+^/CD24^−^ percentage in T-47D cell line and also in CD133^+^ population in sarcoma cell lines (Supplementary Fig. 6C, D). In addition, we observed that cells treated with conditioned media from MAP17-expressing MDA-MB-231 or MDA-MB-468 cells exhibited an increase both in number and size of tumorspheres (Fig. 6d) and in the percentage of holoclones (Fig. 6e). We found that other cells treated with MAP17-conditioned media also exhibited a significant increase in both the number and size of tumorspheres and the percentage of holoclones (Supplementary Fig. 7). In addition, cells treated with MAP17-conditioned media had significantly reduced expression of miR-214, miR-34a, and miR-146a (Fig. 6f).

## Discussion

We report here that MAP17 can regulate miRNA transcription, EMT, and metastasis. MAP17 expression also increases ExVs secretion, being these ExVs enriched on MAP17 and capable of EMT regulation in recipient cells. The regulation of both miRNA transcription and EMT is mediated, at least partially, by the previously described role of MAP17 as a Notch pathway regulator. As a result, MAP17-overexpressing cells increase their tumorigenic properties and are important for metastatic development. In addition, these cells show an increase in stemness phenotypes, characteristics usually connected to metastasis and poor prognosis.

As it has been previously described^1,14,15^, higher MAP17 levels are capable of inducing cell dedifferentiation, resulting in a higher percentage of CSC-like populations. Thus, an increment in MAP17 levels are capable of activating both Notch pathway, through its direct interaction with NUMB protein, and the inflammatory response^15,16^. In addition, we observed how MAP17 levels increase in cancer cells during tumor progression, being higher in metastatic samples than in primary tumor samples, suggesting that this protein could have an important role in progression from primary to metastatic tumors, where this protein usually has higher expression levels^1,8,12,13^. In fact, in breast cancer, MAP17 expression has been correlated with a high risk of disease recurrence^50^. The observed increase in MAP17 expression can be due to progressive MAP17 gene demethylation^1,2,8^, increasing its expression in some of the primary tumor cells. This higher expression has been previously associated with an increase in CSC population through the activation of Notch pathway^15^, which, in fact, increases EMT and the risk of metastasis. A portion of this CSC population can initiate the EMT process, by which epithelial cells are transdifferentiated into motile mesenchymal cells^51,52^. Thus, MAP17 overexpression causes changes in the expression of both cell surface markers and proteins like CDH1, CDH2, and SNAI1, all connected with EMT process. Some cells from primary tumor can exhibit this transition, causing an increment in metastatic potential when MAP17 is overexpressed. Also, the increased secretion of material via ExVs allowed the expansion of the phenotype by horizontal transfer. This horizontal transfer increases the percentage (and risk) of tumor cells that escape from primary tumor to colonize new niches where they subsequently appear as metastases^51–53^.

Focusing on correlations of MAP17 expression with genes connected to tumor progression, we found a high percentage of transcription factors, which suggested a possible pleiotropic role for MAP17 when it is overexpressed in cells. These pleiotropic effects are typical of changes in miRNAs population, owing to the ability of these short non-coding sequences to modify gene expression. Our study allowed us to find a significant number of miRNAs differentially expressed due to MAP17 overexpression. Most of the miRNAs downregulated by MAP17 have been previously described as tumor suppressors, so their decrease would enhance, in many cases, EMT, a typical step required for metastatic development (Supplementary Table 6). In fact, the expression of up to 16 of the identified miRNAs has also been related to a reduction in metastatic potential (Supplementary Table 6). In addition, our data strongly connect MAP17 and miRNA transcription through Notch pathway, with a 60% of the identified miRNAs regulated by MAP17 also connected to Notch pathway. In addition, Notch pathway activation through EGTA treatment in cells with MAP17 overexpression showed lower transcriptional levels of miRNAs, whereas DAPT recovers miRNA expression levels, showing a clear connection between MAP17, Notch pathway and miRNA regulation. In fact, the C-terminal PDZ-binding domain of MAP17 allows it to interact with NUMB, a known binding partner of the NOTCH intracellular domain (NICD), acting as a Notch pathway inhibitor^15^. The breakage of the NUMB-NICD interaction allows NICD translocation to the nucleus, where it forms a ternary complex with CSL and MAML1 to promote the transcription of its target genes^54–56^. Some of the transcribed genes downstream of Notch pathway activation have been connected with the maintaining of self-renewal; those genes include *OCT4*, *NANOG,* and *KLF4*^40,41^ and we have shown here that the downregulation of the miRNAs may play a major role. However, even with DAPT treatment, cells overexpressing MAP17 exhibit lower expression levels for the measured miRNAS, suggesting that, in addition to Notch pathway, other pathways modified by MAP17 could be involved in miRNAs regulation. Thereby, MAP17 overexpression has been previously connected with an increment in ROS and changes in NFκB signaling^9,11,57^. Both processes have been also related to changes in miRNA expression^58,59^.

In the list of genes modulated by miRNA downregulation, we found that some of the miRNAs targets (*SOX2*, *KLF4,* or *NANOG*) were previously described as upregulated owing to MAP17 overexpression^15^. *SOX2*, *KLF4,* and *NANOG*, together with other genes, such as *MYCN*, *KRAS,* or *BRCA1*, which also appeared as targets of some of the identified miRNAs, are transcription factors expressed in CSCs, with critical roles in pluripotency and self-renewal^60,61^. In agreement with these results, we also observed an increase in CSC-like cell population. These results show that MAP17 could modulate changes in cell stemness properties due, at least partly, to changes in miRNAs population.

Cancer cells can modify their microenvironment to obtain advantages that allow their growth^62,63^. ExVs are being recognized as one of the main effectors of these modifications^24–26,64^. We previously showed that MAP17 overexpression induces monocyte differentiation into dendritic cells^16^, so we extended our previous analysis, focusing on the possible dedifferentiation of tumor cells. We found that, through ExVs secretion, MAP17 induces EMT and the acquisition of CSC-like properties. These results demonstrate that MAP17 expression within a cell is not necessary to have a CSC-like phenotype induced by MAP17; exposure to MAP17-loaded ExVs is sufficient. Furthermore, both the specific elimination of ExVs by aldehyde/sulfate latex beads or MAP17 by antibodies from the media, reduced this increase in EMT and stemness, suggesting that MAP17 could be a new target for metastasis in advanced tumors. More work is warranted to prove this initial antimetastatic drug set-up in in vivo models of metastasis or advanced tumors.

All these data, from miRNAs levels to flow cytometry and tumorspheres percentages, are expected in cells forced to overexpress MAP17. To sum up, these results suggest a model in which MAP17 is capable of inducing cell dedifferentiation, both in cells with high MAP17 expression levels and in “target cells”; these activities are mediated, at least partially, by Notch pathway activation. However, more research is needed to evaluate the levels of MAP17 in circulating ExVs and whether these levels are able to induce cell dedifferentiation and metastasis in patients.

Our data highlight not only the role of MAP17 as an important oncogene that regulates through the mechanism of metastasis but also the importance of the horizontal transfer of proteins that induce and/or maintain effective neoplastic or malignant phenotypes.

## Conclusions

In conclusion, increased MAP17 levels in tumors induce multiple changes in gene expression through changes in miRNAs. These changes are dependent on Notch pathway activation. As a consequence, EMT and stemness are induced by increasing the metastatic potential of these cells both in vitro and in vivo. In addition, MAP17 overexpression also increased ExVs secretion, including secretion of itself, inducing EMT and CSC phenotypes by horizontal transference. Therefore, MAP17 expression enhances the horizontal propagation of EMT and metastatic phenotypes by transferring MAP17 protein between subsets of neoplastic cells. Finally, the elimination of MAP17 by antibodies from the media, reduces this increase in EMT and stemness, suggesting that MAP17 could be a new target for metastasis in advanced tumors.

## Supplementary information

Supplementary Material, Tables and Figures
