# Supplementary material for: Breast tumor cells promotes the horizontal propagation of EMT, stemness, and metastasis by transferring the MAP17 protein between subsets of neoplastic cells
Source: Oncogenesis. 2020 Oct 26;9(10):96. doi: 10.1038/s41389-020-00280-0 (PMC7589521; doi:10.1038/s41389-020-00280-0)
Supplement: Supplementary file 1 — Supplementary Material, Tables and Figures [file 41389_2020_280_MOESM1_ESM.docx]

**Supplementary Material**

**Material and Methods**

**Bioinformatics analysis.**

To find genes correlated with *MAP17* expression, we selected 31 breast cancer databases (**see Supplementary Table 1**), all freely accessible through R2 webpage (http://r2.amc.nl). We used two different gene filters: Oncogenesis (GeneCategory) and Pathways in Cancer (KEGG Pathway); both options included in R2. We searched for correlations using the *MAP17* probes listed in **Supplementary Table 1**, establishing a p-value lower than 0.05 to identify significant differences. From the list of correlated genes, we separated genes positively from genes negatively correlated with *MAP17* expression, generating two gene lists for each database.

To look for altered biological processes connected to changes in *MAP17* expression, we used enrichment analysis from Gene Ontology consortium webpage (http://geneontology.org/page/go-enrichment-analysis). The obtained GO terms, from genes that were either positively or negatively correlated with MAP17 expression, were compared using Venny tool[^34^](#_ENREF_34). Additionally, we used Panther (http://www.pantherdb.org/) to group the list of genes according to protein class.

TransmiR v2.0 software (http://www.cuilab.cn/transmir) was used to find miRNAs regulated by NOTCH1, HES1 or HES5.

Datasets GSE20685 and GSE7390 were used to separate patients according to tumor type (primary vs metastasis) and *MAP17* levels (low vs high), using GEO2R (<https://www.ncbi.nlm.nih.gov/geo/geo2r/>) to obtain the expression values of each individual gene.

**Cell lines and cellular assays.**

T-47D, MDA-MB-231, MDA-MB-468 and MCF10A cells were obtained from the European Collection of Authenticated Cell Cultures (ECACC) commercial repository at the beginning of this study. No further authentication was performed in these cell lines. AA, AW, AX, BC and CE cell lines, derived from sarcoma patients, were described previously[^35^](#_ENREF_35). T-47D, MDA-MB-231 and MDA-MB468 cells were maintained in DMEM (Gibco), while sarcoma cells were maintained in F10 (Gibco), all supplemented with 10% FBS (Life Technologies), penicillin, streptomycin, and fungizone. All cell lines were regularly tested for mycoplasma. MAP17 expression was induced through transfection with plasmid pBabe-MAP17, previously described[^12^](#_ENREF_12)^,^[^15^](#_ENREF_15). All transfected cells were selected with 1 μg mL^-1^ of puromycin. Clonogenicity assays, holo- and paraclone analysis and tumorspheres analysis were performed as previously described[^36^](#_ENREF_36).

**miRNAs analysis.**

We extracted total RNA from T-47D cells, overexpressing MAP17 or control, using Qiazol and miRNAeasy kit (Qiagen, USA). To find miRNAs with significant differences between both conditions, we used the Cancer Pathway Finder miScript miRNA PCR Array (Qiagen, USA), following manufacturer’s instructions. All miRNAs detected with significant differences were analyzed using miRTarBase resource (miRTarBase.mbc.nctu.edu.tw/), focusing only in changes in gene expression detected by direct Reporter Assay or Western Blot.

**Analysis of gene transcription.**

Total RNA was purified as described previously[^15^](#_ENREF_15)^,^[^36^](#_ENREF_36). To detect changes in gene expression, we used the probes listed in **Supplementary Table 2**. From the list of miRNAs with significant changes, we selected five of them, also listed in **Supplementary Table 2**. All probes were purchased from Life Technologies and retro-transcribed following manufacturer’s instructions.

Quantitative PCR was performed as described previously[^15^](#_ENREF_15)^,^[^36^](#_ENREF_36). At least three independent experiments in triplicate samples were performed for each analyzed gene. Student’s t-test was applied for each pair of samples, with a significance threshold of p<0.05.

**Protein extraction and WB analysis.**

Protein extracts for WB analysis were obtained as described previously[^36^](#_ENREF_36), with the exception of cell extracts used for MAP17 detection, where RIPA buffer included 6M urea. For WB detection, we used anti-MAP17 monoclonal antibodies, anti-SNAI1 (Cell Signaling, C15D3), anti-CDH1 (Santa Cruz Biotechnology, sc-8426), anti-CDH2 (Santa Cruz Biotechnology, sc271386), anti-CD63 (Thermofisher #10628D) and anti-calnexin (Santa Cruz, sc-23954). α-Tubulin (T9026, Sigma) was used as a control. Horseradish peroxidase-labeled rabbit anti-mouse (ab97046, Abcam) and goat anti-rabbit (ab97051, Abcam) secondary antibodies were used.

**Fluorescence-activated cell sorting (FACS) analysis.**

MDA-MB-231, MDA-MB-468, T-47D, AA, AW, AX, BC and CE cells were washed once with PBS and harvested with 0.05% trypsin/0.025% EDTA. Detached cells were centrifuged and resuspended in wash buffer (PBS, 2% FBS, 5 mM EDTA). One million cells was resuspended in 125 μL of this buffer and blocked for 10 minutes with 12.5 μL of FcR Blocking Reagent (MACS MiltenyiBiotec, 130-059-901). Combinations of fluorochrome-conjugated monoclonal antibodies from MACS MiltenyiBiotec against human CD44 (APC; 130-095-177), CD24 (PE; 130-095-953); CD63 (APC; 130-100-182), CD105 (APC; 130-099-125) or CD133 (PE; 130-098-826) were added to the cell suspension following manufacturer’s instructions and incubated at 4°C in the dark for 30 minutes. Labeled cells were washed twice with wash buffer, resuspended in 300 μL of wash buffer and analyzed on a FACS Canto II Analyzer cytometer.

**Cell migration and invasion assay.**

Cells (2.5×10^5^) were seeded in Boyden chambers with 8.0 µm pore size (Nunc, Thermo Fisher) in serum-free medium. Medium containing 10% FBS served as a chemoattractant in the lower chamber. After 8 h, cells were fixed with glutaraldehyde 0.5% and stained with crystal violet 1%. Next, non-invading cells were removed with cotton swabs. Ten microscopic fields of invading cells were counted for each well. Data are represented as the mean ± SEM from three individual experiments.

**Mouse luciferase assay.**

To determine whether MAP17 increases metastatic potential, MDA-MB-231 cells, previously transfected with pBabe-EV or pBabe-M17, were infected with plasmid pLenti-II-CMV-Luc2-IRES-GFP, that allow expression of both luciferase and GFP, and GFP^+^ cells were selected by flow cytometry. Three million cells from each cell line were injected into the mammary fat pad of three 6-week-old Foxn1^nu^ athymic nude female mice (Harlan Laboratories, Netherlands), with no randomization nor researcher blinding. When primary tumors reached a size of 10 mm^2^, tumors were surgically removed and mice continually assessed for tumor recurrence. To analyze the appearance of metastatic tumors, a luciferin solution (XenoLight D-luciferin-K^+^ salt bioluminescent substrate, PerkinElmer, 122799) was injected at a final concentration of 150 mg luciferin/kg of mouse body weight. Mice were anesthetized with inhaled isoflurane 10 minutes after luciferin injection. To detect GFP fluorescence, mice were analyzed 15 minutes after luciferin injection in an IVIS Lumina Series III (Perkin Elmer). Then, mice were euthanized with CO_2_, and their organs were also visualized. In all cases, the optimal exposure time was determined by the software (Living Image 4.5.4).

**Treatment with conditioned media.**

Conditioned media from transfected MDA-MB-231 and MDA-MB-468 cells were obtained and used as previously described[^16^](#_ENREF_16). Non-transfected MDA-MB-231, MDA-MB-468, T-47D, AA, AW, AX, BC and CE cells were seeded in 6-well plates and allowed to grow for 24 hours. After that, media was substituted by a 1:1 conditioned media:fresh medium, and cells grow for other 48 hours before total RNA was extracted to evaluate changes in miRNAs, gene expression and surface cell markers, as described above. Additionally, in order to evaluate if vesicles derived from conditioned media were responsible for cell dedifferentiation, 4 mL of conditioned media were incubated with 5 μL of Aldehyde/Sulfate Latex Beads 4% w/v (Thermo Fisher, A37304) under stirring for 2 hours. After that, media was centrifuged at 4000 rpm for 5 minutes, using supernatant to treat MDA-MB-231 and MDA-MB-468 cells for 48 hours, in a proportion 1:1 with fresh medium. For tumorsphere assay, a total of 10^5^ cells were cultured in Ultra-Low Attachment Multiwell Plates (Corning) in triplicate, and tumorspheres were counted 5 days after seeding.

Additionally, 4 mL of conditioned media were incubated with 100 μL of polyclonal antibodies against MAP17 and 10 μL of protein A-sepharose or only with 10 μL of protein A-sepharose under stirring for 2 hours at 4 ºC and centrifuged as above. As a control, 4 mL of conditioned media, previously treated with 5 μL of Aldehyde/Sulfate Latex Beads 4% w/v for 2 hours and centrifuged as above, were incubated also with 100 μL of polyclonal antibodies against MAP17 and 10 μL of protein A-sepharose. All conditioned media were used for treating MDA-MB-231 cells for 48 hours and total RNA was extracted as described above.

**ExVs isolation and detection.**

Conditioned media from MDA-MB-231 or MDA-MB-468 cells were centrifuged for 30 min at 10000 g at 4°C, transferred to a new tube, equilibrated with TBS-Ca^2+^ and centrifuged 2 hours at 100000 g at 4°C. Then, the supernatant (SN) was discarded, TBS-Ca^2+^ was added again, and the sample was centrifuged for 1 hour at 100000 g and 4 °C. Finally, the SN was discarded by decantation, being the pellet (considered to be an ExVs enriched fraction) resuspended in RIPA buffer for detection by WB.

To detect ExVs fusion in cells, MDA-MB-231 or MDA-MB-468 cells (EV or MAP17) overexpressing cytoplasmic GFP were cultured for 72 hours, and ExVs were purified as indicated above. Non-transfected MDA-MB-231 cells, seeded in 6-well plates, were used to detect possible ExVs in cells. For that purpose, an ExVs-enriched fraction, resuspended in TBS-Ca^2+^ buffer, was added to the cells and incubated at 37°C, 5% CO_2_ for 90 minutes. After that, cells were visualized using an Olympus BX61 fluorescence microscope.

For FACS analysis of ExVs, 3.5x10^5^ MDA-MB-231 or MDA-MB-468 cells were seeded at 6 cm^2^ plates with 3 mL of DMEM and cultured for 48 hours. Then, cell media was removed and centrifuged for 30 min at 10000 g at room temperature, being the supernatant incubated with 2 µL of Aldehyde/Sulfate Latex Beads 4% w/v (Thermo Fisher, A37304) for 30 min at room temperature in a rotator mixer. Finally, the beads were recovered by centrifugation at 4000 g for 5 minutes, washed twice with PBS, and labeled with CD63 antibody using the conditions described above.

**Competing interests:** The authors declare that they have no competing interests

**Authors' contributions:** JMG-H and AC conceived and designed this study. JMG-H, DO-A, MP, EPC, SM-G performed the experiments; JMG-H and AC analyzed and interpreted the data, and drafted the manuscript. All authors revised the manuscript.

**Availability of supporting data.**

No datasets were generated during the current study. The datasets analyzed during the current study are available in the different repositories already mentioned in the Methods section.

| **Supplementary Table 1**. List of breast tumoral datasets used in this work | | | |
| --- | --- | --- | --- |
| Dataset | *MAP17* probe | Algorithm | Identifier |
| Hofman | 219630_at | MAS5.0 | GSE3307 |
| Roth | 219630_at | MAS5.0 | GSE7307 |
| Roth | 219630_at | MAS5.0 | GSE3526 |
| Su | 219630_at | MAS5.0 | GSE1133 |
| Bergh | 219630_at | MAS5.0 | GSE1456 |
| Bertucci | 219630_at | MAS5.0 | GSE21653 |
| Black | 219630_at | MAS5.0 | GSE36771 |
| Booser | 219630_at | MAS5.0 | GSE25066 |
| Bos | 219630_at | MAS5.0 | GSE12276 |
| Brown | 219630_at | MAS5.0 | GSE76124 |
| Chin | 219630_at | MAS5.0 | GSE69031 |
| Concha | 219630_at | MAS5.0 | GSE29431 |
| Desmedt | 219630_at | MAS5.0 | GSE16391 |
| EXPO | 219630_at | MAS5.0 | GSE2109 |
| Halfwerk | 219630_at | Complex | ps_avgpres_breasthalfwerk947_u133a |
| Harris | ILMN_1708580 | Custom | GSE76360 |
| Iglehart | 219630_at | MAS5.0 | GSE5460 |
| Iwamoto | 219630_at | MAS5.0 | GSE22093 |
| Jonsdottir | ILMN_1708580 | Custom | GSE46563 |
| Loi | 219630_at | MAS5.0 | GSE9195 |
| Meijers-Heijboer | 219630_at | MAS5.0 | GSE27830 |
| Miller | 219630_at | MAS5.0 | GSE3494 |
| Minn | 219630_at | MAS5.0 | GSE2603 |
| Plaut | 219630_at | MAS5.0 | GSE10797 |
| Prat | 219630_at | MAS5.0 | GSE50948 |
| Quiles | 219630_at | MAS5.0 | GSE28844 |
| Servant | ILMN_1708580 | Custom | GSE30682 |
| Sinn | 219630_at | MAS5.0 | GSE124648 |
| Smid | 219630_at | MAS5.0 | GSE29271 |
| Sotiriou | 219630_at | MAS5.0 | GSE7390 |
| TCGA528 | PDZK1IP1 | Custom | ps_avgpres_tcgabrca528_tcgaovag1 |
| TCGA1097 | PDZK1IP1_10158 | Rsem | ps_avgpres_tcgabrca1097_tcgars |
| Wang | 219630_at | MAS5.0 | GSE2034 |
| Wessels | ILMN_1708580 | Custom | GSE34138 |
| Yu | 219630_at | MAS5.0 | GSE102484 |
| Zhang | 219630_at | MAS5.0 | GSE12093 |

| **Supplementary Table 2**. List of probes used in this work | | | | |
| --- | --- | --- | --- | --- |
| Gene | Code |  | miRNA | Code |
| *MAP17* | Hs00906696_m1 |  | hsa-miR-214 | ID 002306 |
| *CTGF* | Hs01026927_g1 |  | hsa-miR-34a | ID 000426 |
| *NFKB1* | Hs00765730_m1 |  | hsa-miR-146a | ID 000468 |
| *NOTCH1* | Hs01062014_m1 |  | hsa-miR-205 | ID 000509 |
| *NOTCH2* | Hs01050702_m1 |  | hsa-miR-18a | ID 002422 |
| *PIM1* | Hs01065498_m1 |  | hsa-miR-RNU48* | ID 001006 |
| *FOXC2* | Hs00270951_s1 |  |  |  |
| *SNAI1* | Hs00195591_m1 |  |  |  |
| *TWIST1* | Hs01675818_s1 |  |  |  |
| *VIM* | Hs00958111_m1 |  |  |  |
| *OCT4* | Hs00999632_g1 |  |  |  |
| *SOX2* | Hs01053049_s1 |  |  |  |
| *KLF4* | Hs00358836_m1 |  |  |  |
| *NANOG* | Hs04260366_g1 |  |  |  |
| *HES1* | Hs00172878_m1 |  |  |  |
| *ACTB** | Hs01060665_g1 |  |  |  |
| *Housekeeping probe | | | | |

| **Supplementary Table 3.** List of genes connected to tumorigenesis or altered pathways in cancer positively or negatively correlated with *MAP17*. | | | | | | |
| --- | --- | --- | --- | --- | --- | --- |
| Positive correlations | | | | Negative correlations | | |
| *ABL2* | *EIF4EBP1* | *IRF1* | *PLXDC2* | *ADCY1* | *FRAT1* | *MSH3* |
| *ADCY2* | *EIF4EBP2* | *IRF6* | *PML* | *ADCY5* | *GLI3* | *MST1* |
| *ADCY3* | *ELF3* | *ITGA2* | *PPARD* | *ADCY6* | *GNA13* | *NCOA3* |
| *ADCY7* | *ELL2* | *ITGA6* | *PPP4C* | *ADCY9* | *GNAQ* | *PBX1* |
| *AKT3* | *EML4* | *ITGAV* | *PRKCA* | *AFF1* | *GNB1* | *PDGFB* |
| *ALK* | *EMP1* | *JAK1* | *PROX1* | *AFF4* | *GNB5* | *PDGFRB* |
| *ARAF* | *ENG* | *JAK2* | *PTCH1* | *AGTR1* | *GNG10* | *PGR* |
| *ARHGEF11* | *EPAS1* | *JAK3* | *PTGS2* | *AKT2* | *GNG13* | *PHF15* |
| *BAK1* | *ETS1* | *JUN* | *RAC2* | *APC* | *GNGT1* | *PLCG1* |
| *BCL2L14* | *ETS2* | *JUNB* | *RAET1G* | *AR* | *GRB2* | *PLXDC1* |
| *BCR* | *ETV4* | *KIT* | *RALB* | *ARNT2* | *GSTM3* | *POLK* |
| *BID* | *ETV5* | *LAMB3* | *RARB* | *ASCL1* | *GSTO2* | *PPP2CA* |
| *BIRC2* | *ETV6* | *LAMC2* | *RASSF5* | *AXIN2* | *HDAC1* | *PTEN* |
| *BIRC3* | *ETV7* | *LCN2* | *RBX1* | *BCL2* | *HEYL* | *PTGER2* |
| *BMP2* | *FAS* | *LMO1* | *RELA* | *BDKRB2* | *HRAS* | *PTGER3* |
| *BMP4* | *FBLIM1* | *LMO4* | *RGR* | *BECN1* | *HSP90AA1* | *PTK2* |
| *BTG1* | *FES* | *LOXL4* | *RRAS* | *BRAF* | *HSP90AB1* | *RARA* |
| *CALML4* | *FGF2* | *LPAR2* | *RRAS2* | *BRCA1* | *IGF1R* | *RBL2* |
| *CALML5* | *FGFR2* | *LPAR3* | *RUNX3* | *BRIP1* | *IKBKB* | *RET* |
| *CASP8* | *FGR* | *LPP* | *S100A11* | *BTG2* | *IL6ST* | *RHOH* |
| *CBFB* | *FH* | *LRP5* | *S100A3* | *CALM1* | *ING2* | *RHOT2* |
| *CBL* | *FRAT2* | *LYN* | *SHC1* | *CAMK2B* | *ITGA2B* | *RNF4* |
| *CCDC6* | *FRK* | *MAGEA4* | *SKI* | *CCND1* | *ITGA3* | *RPS6KB1* |
| *CCNA1* | *FSCN1* | *MAGEB1* | *SKP2* | *CDKN1A* | *JUND* | *RUNX1T1* |
| *CCND3* | *FZD1* | *MAGED1* | *SMO* | *CDKN1B* | *KITLG* | *RXRA* |
| *CCNE1* | *FZD10* | *MAPK1* | *SOS1* | *CTBP1* | *LAMA3* | *SEMA3B* |
| *CDK2AP1* | *FZD7* | *MCL1* | *SPI1* | *CTNNA1* | *LAMA5* | *SKP1* |
| *CDK2AP2* | *FZD9* | *MET* | *ST5* | *CUL3* | *LAMB1* | *SMAD3* |
| *CDK6* | *GADD45A* | *MGST1* | *STAT2* | *E2F1* | *LMO3* | *SMAD5* |
| *CDKN2A* | *GIPC1* | *MGST3* | *STAT3* | *EDNRA* | *LOXL1* | *SMAD7* |
| *CHEK1* | *GNAI1* | *MIA* | *STAT5A* | *EPOR* | *MAGED2* | *STK4* |
| *CITED2* | *GNB4* | *MLF2* | *TACC2* | *ERBB3* | *MAGEE1* | *SYNPO2* |
| *CITED4* | *GNG12* | *MME* | *TCF7L1* | *ERBB4* | *MAGEF1* | *SYNPO2L* |
| *CKS1B* | *GNG5* | *MMP1* | *TCF7L2* | *ESR1* | *MAGEH1* | *TACC1* |
| *COL4A3* | *GSTA1* | *MSH2* | *TES* | *F2R* | *MAPK3* | *TFAP2A* |
| *COL4A4* | *GSTA2* | *NCOA1* | *TFAP2B* | *FADD* | *MAX* | *TGFB3* |
| *CRK* | *GSTA4* | *NFE2L2* | *TFG* | *FER* | *MCF2L* | *TRAF4* |
| *CSF1R* | *GSTP1* | *NFKB1* | *TGFA* | *FGF18* | *MDM2* | *TSC1* |
| *CSF2RA* | *HDAC2* | *NFKB2* | *TGFB2* | *FGFR1* | *MDM4* | *USP6* |
| *CSF2RB* | *HES1* | *NFKBIA* | *TGFBR1* | *FGFR3* | *MEIS3* | *VAV2* |
| *CTNNB1* | *HIF1A* | *NKX3-1* | *TRAF1* | *FGFR4* | *MLH1* | *VEGFC* |
| *CUL1* | *IFNAR2* | *NOTCH1* | *TRAF3* | *FLT3* | *MLH3* |  |
| *CXCL8* | *IFNGR1* | *NOTCH2* | *TRAF6* |  |  |  |
| *CYR61* | *IFNGR2* | *NUP98* | *TYRO3* |  |  |  |
| *DAPK1* | *IGF2R* | *OAZ3* | *ULBP2* |  |  |  |
| *DAPK2* | *IL12A* | *OGFRL1* | *USP6NL* |  |  |  |
| *DDX26B* | *IL12RB2* | *PCBP4* | *VAV1* |  |  |  |
| *DENND2C* | *IL15* | *PIK3CB* | *VEGFA* |  |  |  |
| *DMBT1* | *IL15RA* | *PIK3CD* | *WNT10A* |  |  |  |
| *E2F3* | *IL2RA* | *PIK3R1* | *WNT5A* |  |  |  |
| *EGF* | *IL4R* | *PIK3R3* | *WNT6* |  |  |  |
| *EGFR* | *IL6* | *PIM1* | *WT1* |  |  |  |
| *EGLN1* | *IL6R* | *PIM2* | *YES1* |  |  |  |
| *EGLN3* | *IL7* | *PLCG2* |  |  |  |  |
| *EI24* | *IL7R* | *PLD1* |  |  |  |  |
|  |  |  |  |  |  |  |

**SUPPLEMENTARY FIGURES**


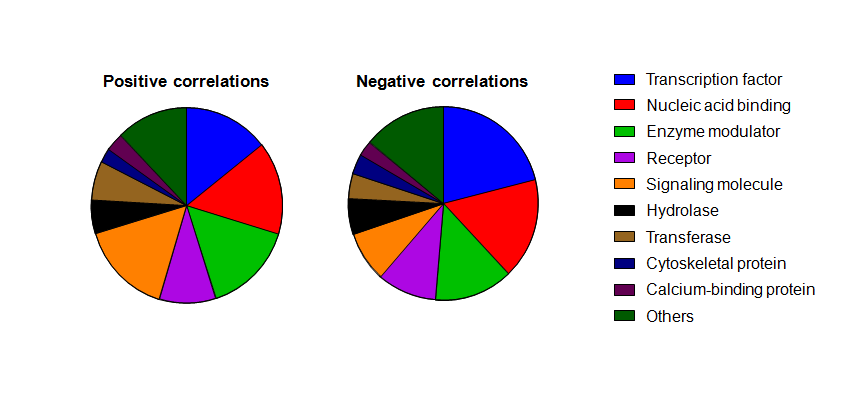


**Supplementary Figure 1.** Pie chart of positive or negative gene correlations with MAP17, according protein class Panther classification system.


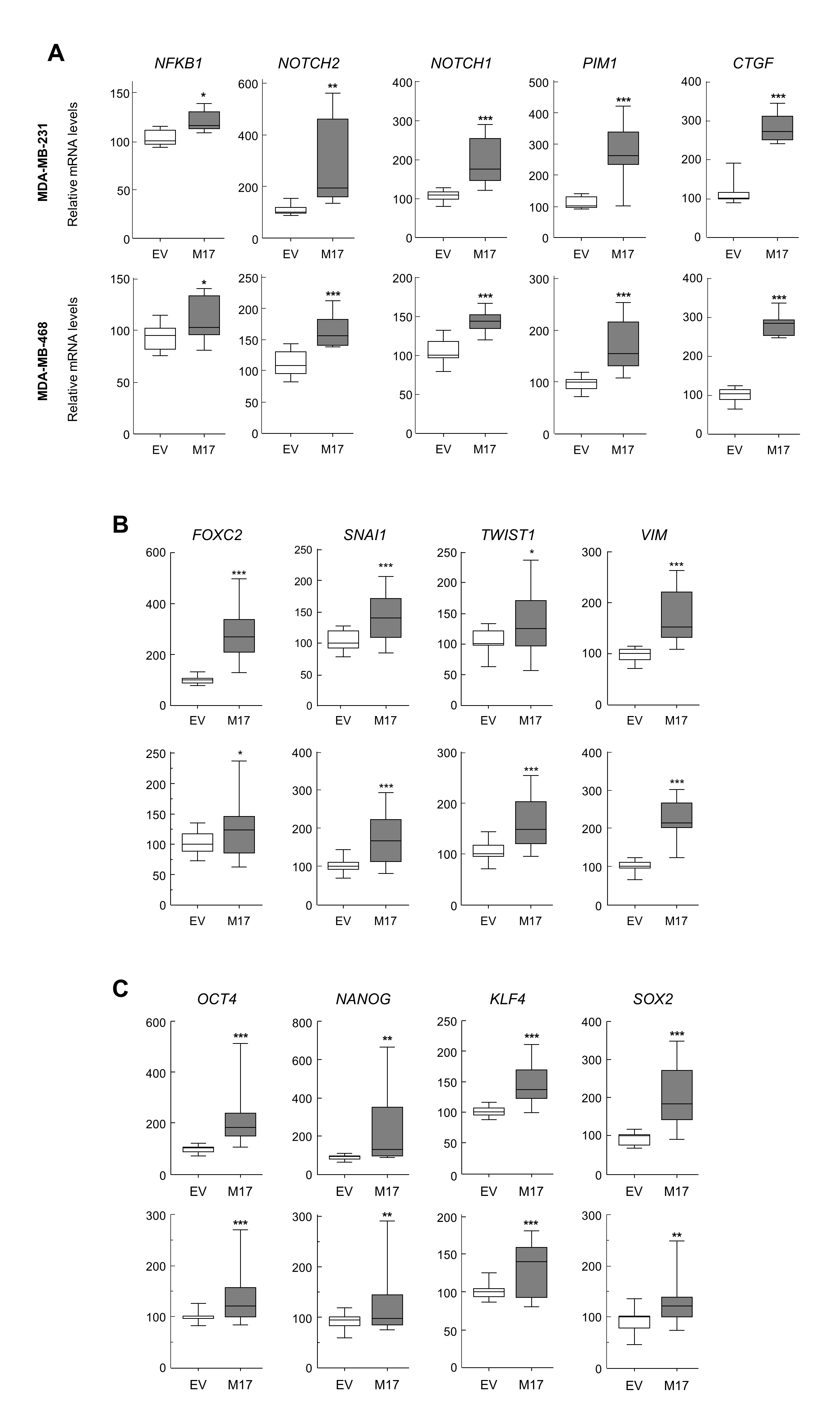


**Supplementary Figure 2. A)** qPCR analysis of genes targeted by miRNAsdownregulated due to MAP17 overexpression. **B)** qPCR analysis of EMT-related genes in MDA-MB-231 and MDA-MB-468 cells. **C)** qPCR analysis of stem cell genes in MDA-MB-231 and MDA-MB-468 cells. In general, MAP17 overexpression causes a significant increment in mRNA levels of the measured genes.


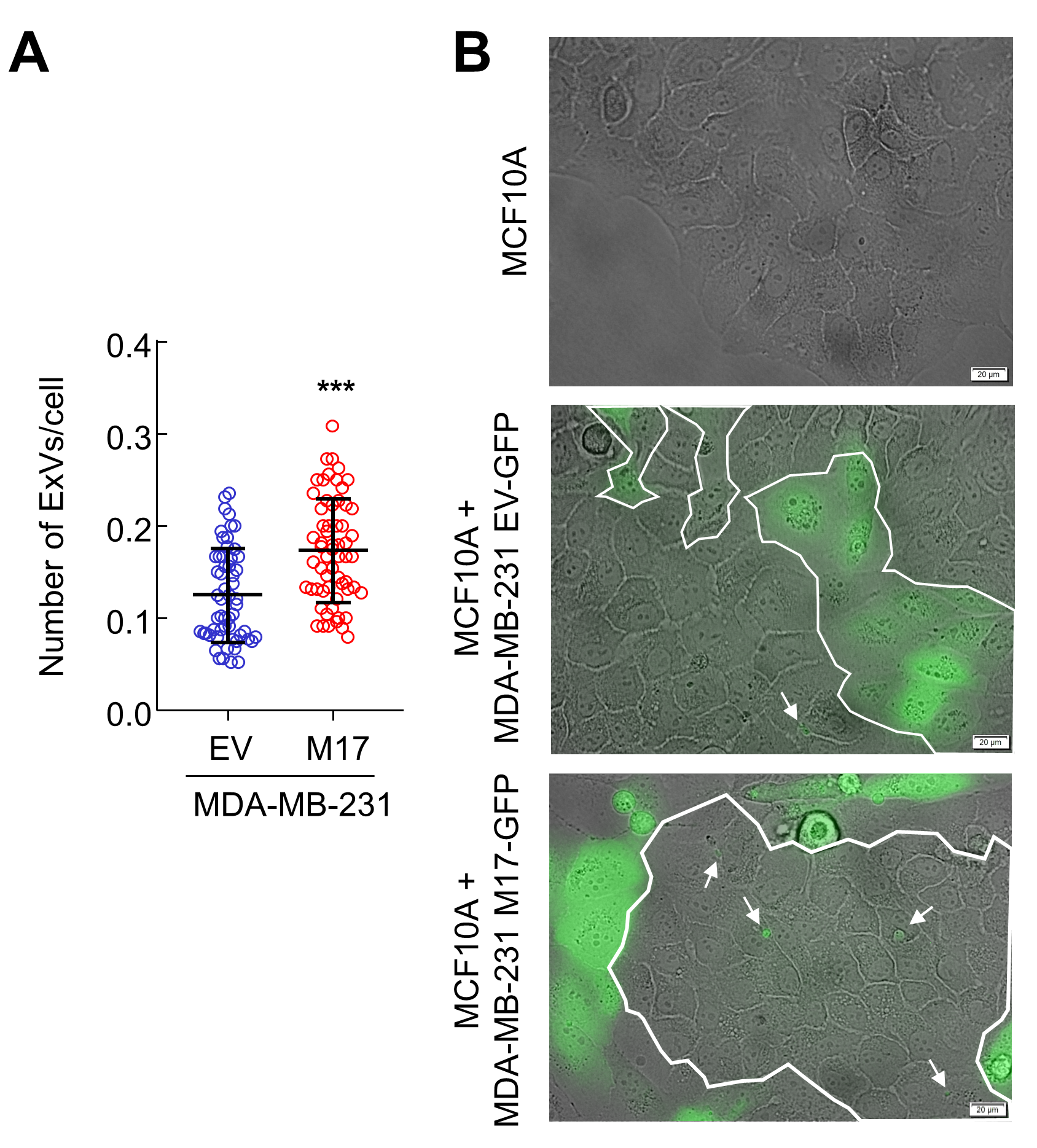


**Supplementary Figure 3. A)** Number of ExVs detected by fluorescent cells in, at least, 10 different images. **B)** Fluorescent microscopy images of MCF10A alone, cocultured with MDA-MB-231 cells (EV or M17) overexpressing GFP Fluorescent cells correspond to MDA-MB-231 cells, while white arrows point to possible ExVs in MCF10A cells.


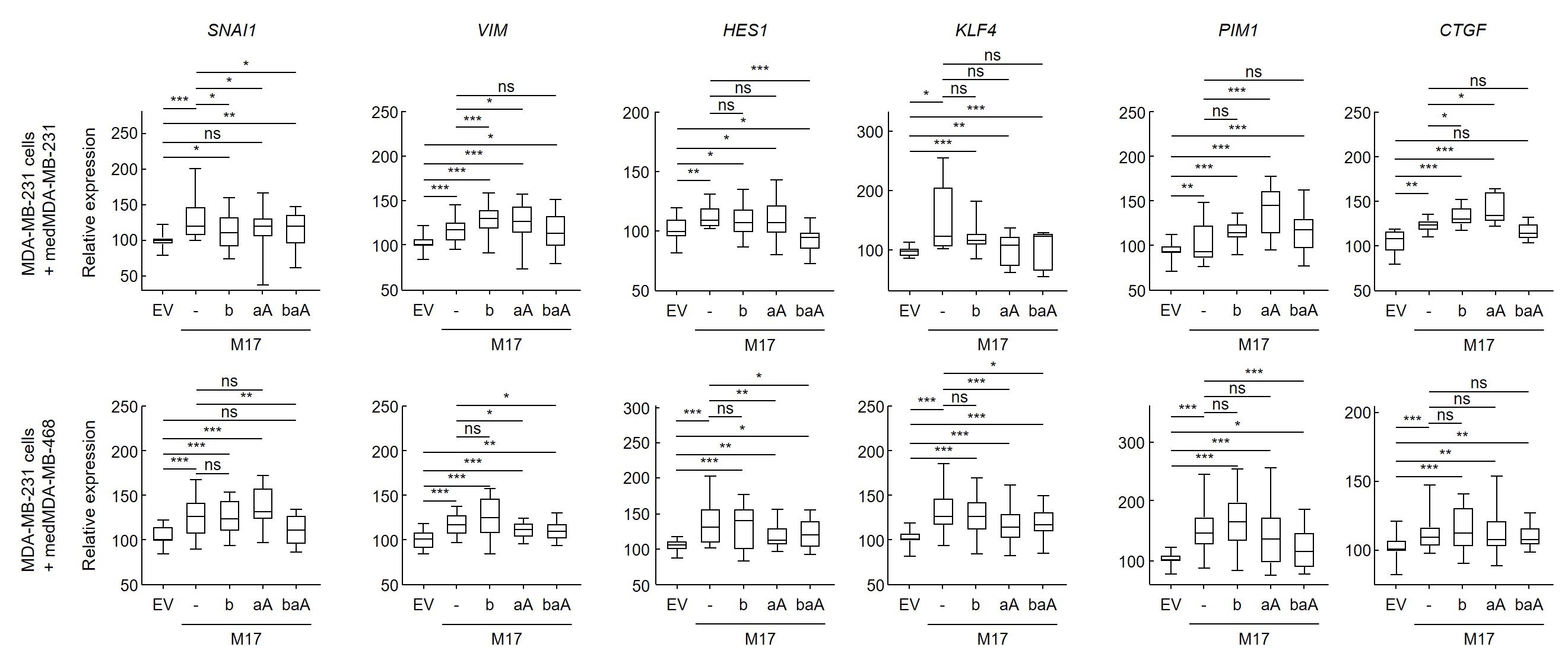


**Supplementary Figure 4.** MAP17 causes an increment in cell dedifferentiation in target cells due to ExVs secretion. MDA-MB-231 cells were incubated with conditioned media from MDA-mB-231 or MDA-MB-468 cells, overexpressing MAP17 (M17) or not (EV). Conditioned media was treated with aldehyde/sulfate latex beads (b) to capture ExVs, with protein A/Sepharose + antiMAP17 (aA) to capture MAP17 or with both aldehyde/sulfate latex beads and protein A/Sepharose + antiMAP17 (baA).


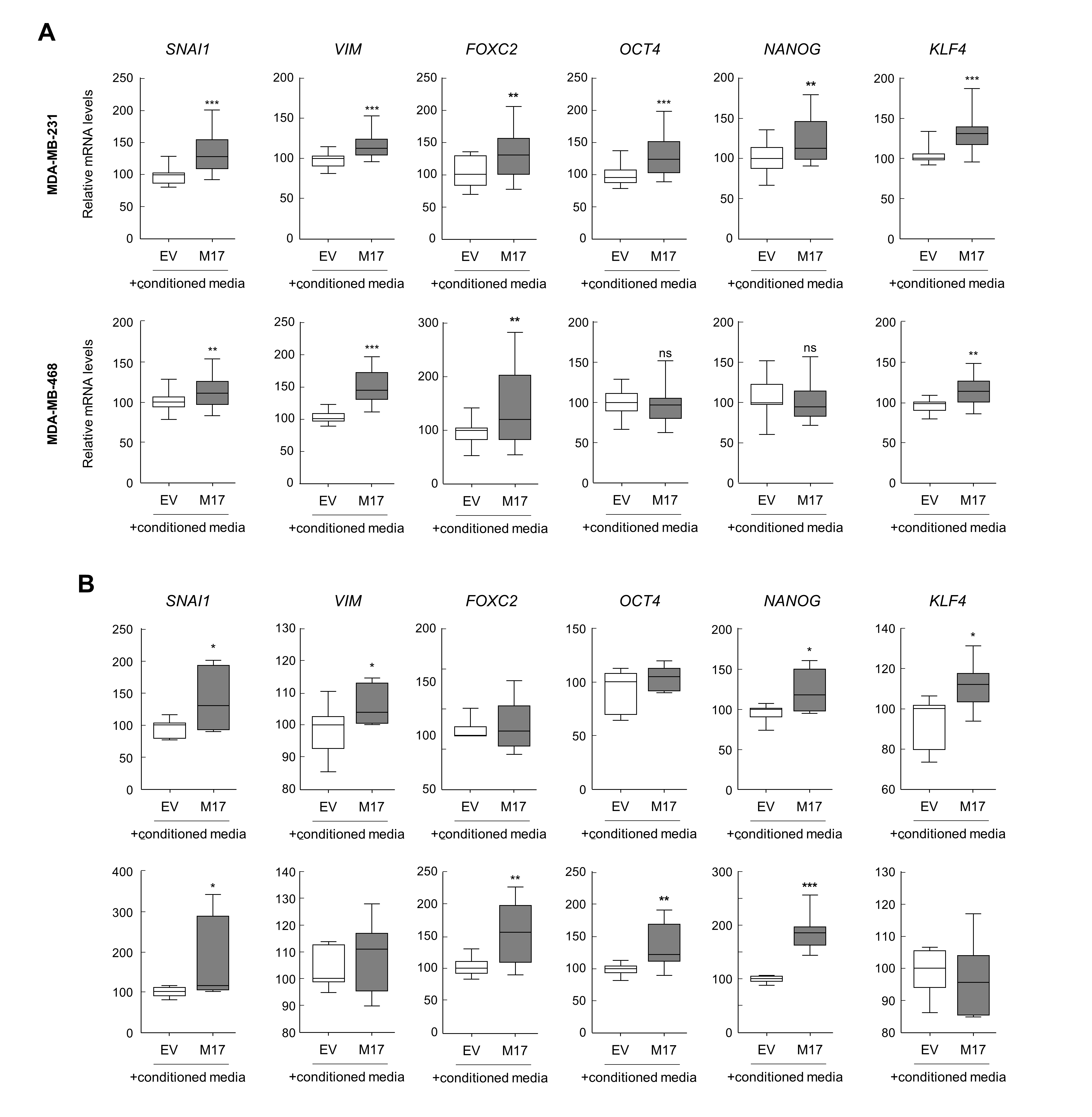


**Supplementary Figure 5. A)** qPCR analysis of stem cell and EMT-related genes in MDA-MB-231 and MDA-MB-468 cells treated with MDA-MB-231 conditioned media. **B)** qPCR analysis of stem cell and EMT-related genes in MDA-MB-231 and MDA-MB-468 cells treated with MDA-MB-468 conditioned media. Student t-test statisticalanalysis of the data was performed to find statistical differences (*, p< 0.05; **, p< 0.01; ***, p< 0.001). EV, empty vector; M17, MAP17.


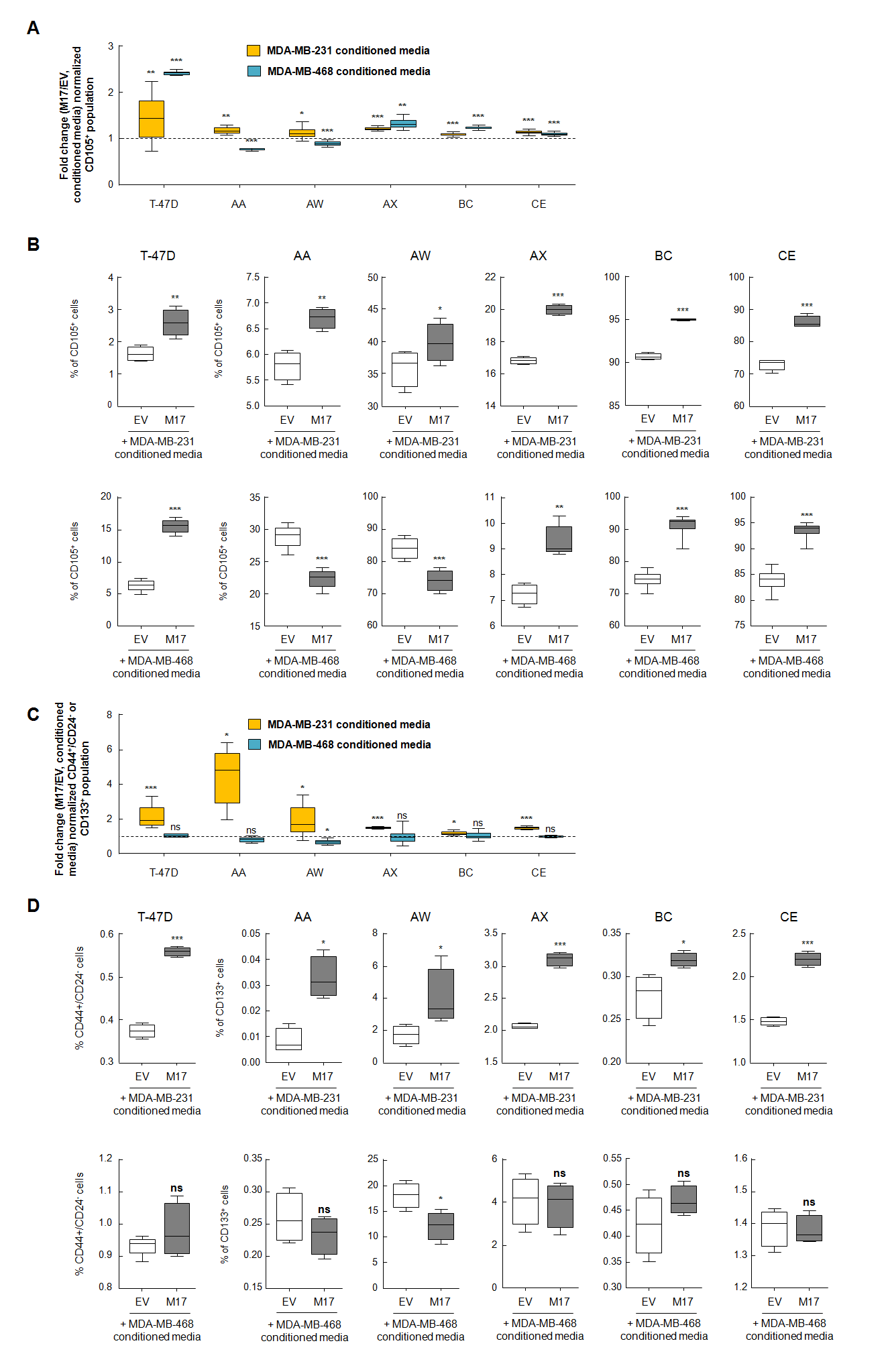


**Supplementary Figure 6. A)** Fold change of the CD105^+^ subpopulation for T-47D, AA, AW, AX, BC and BG sarcoma cells, treated with MDA-MB-231 or MDA-MB-468 conditioned media, measured by analytic FACS. **B)**Analytic FACS to identifythe CD105^+^ subpopulation in T-47D, AA, AW, AX, BC and BG cells treated with MDA-MB-231 or MDA-MB-468 conditioned media. **C)** Fold change of the CD44^+^/CD24^-^subpopulation for T-47D or CD133^+^ subpopulation for AA, AW, AX, BC and BG sarcoma cells, treated with MDA-MB-231 or MDA-MB-468 conditioned media, measured by analytic FACS. **D)**Analytic FACS to identifythe CD44^+^/CD24^-^ subpopulation in T-47D cells or CD133^+^ subpopulation in AA, AW, AX, BC and BG sarcoma cells treated with MDA-MB-231 or MDA-MB-468 conditioned media. Student t-test statisticalanalysis of the data was performed to find statistical differences (*, p< 0.05; **, p< 0.01; ***, p< 0.001). EV, empty vector; M17, MAP17.


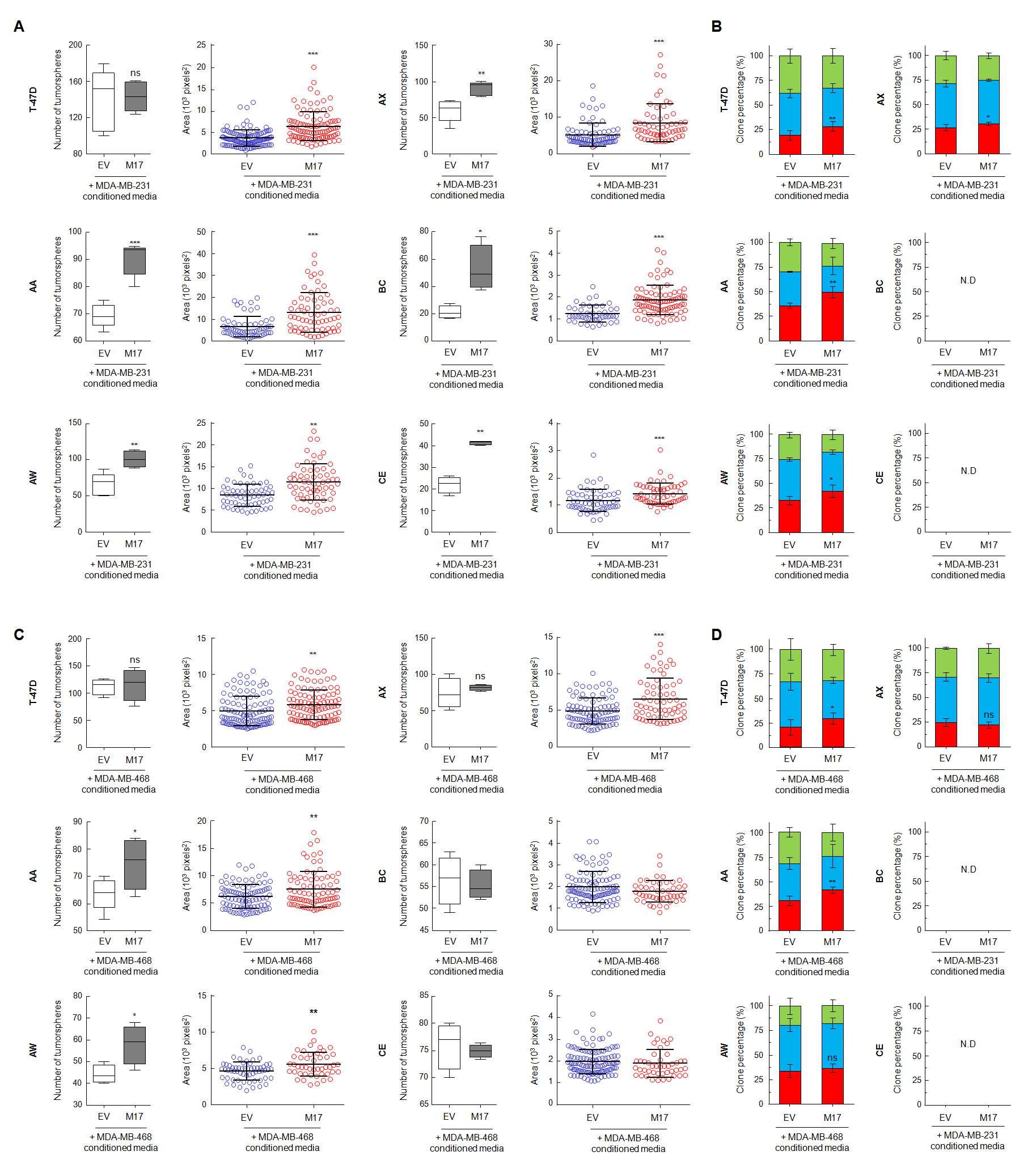


**Supplementary Figure 7. A)** Number and area of tumorspheres in cells treated with conditioned media derived from MDA-MB-231 cells. **B)** Percentage of holoclones, meroclones and paraclonesin cells treated with conditioned media derived from MDA-MB-231 cells. For BC and CE, clones percentage could not be determined due to the disperse growth of the cells, that did not allow to distinguish individual clones. **C)** Number and area of tumorspheres in cells treated with conditioned media derived from MDA-MB-468 cells. **D)** Percentage of holoclones, meroclones and paraclonesin cells treated with conditioned media derived from MDA-MB-468 cells. For BC and CE, clones percentage could not be determined due to the disperse growth of the cells, that did not allow to distinguish individual clones. Student t-test statistical analysis of the data was performed to find statistical differences (*, p < 0.05; **, p < 0.01; ***, p < 0.001). EV, empty vector; M17, MAP17.
